# Boosting nanomedicine performance by conditioning macrophages with methyl palmitate nanoparticles†

**DOI:** 10.1039/d1mh00937k

**Published:** 2021-07-29

**Authors:** Roberto Palomba, Martina di Francesco, Valentina di Francesco, Federica Piccardi, Tiziano Catelani, Miguel Ferreira, Anna Lisa Palange, Paolo Decuzzi

**Affiliations:** Laboratory of Nanotechnology for Precision Medicine – Fondazione Istituto Italiano di Tecnologia Via Morego 30 16163 Genova Italy paolo.decuzzi@iit.it; Department of Informatics, Bioengineering, Robotics and System Engineering, University of Genoa Via Opera Pia, 13 Genoa 16145 Italy; Animal Facility – Fondazione Istituto Italiano di Tecnologia Via Morego 30 16163 Genova Italy; Electron Microscopy Facility – Fondazione Istituto Italiano di Tecnologia Via Morego 30 16163 Genova Italy

## Abstract

Surface PEGylation, biological camouflage, shape and stiffness modulation of nanoparticles as well as liver blockade and macrophage depletion have all improved the blood longevity of nanomedicines. Yet, the mononuclear phagocytic system still recognizes, sequesters, and processes the majority of blood borne particles. Here, the natural fatty acid methyl palmitate is combined with endogenous blood components – albumin – realizing ∼200 nm stable, spherical nanoparticles (MPN) capable of inducing a transient and reversible state of dormancy into macrophages. In primary bone marrow derived monocytes (BMDM), the rate of internalization of 5 different particles, ranging in size from 200 up to 2000 nm, with spherical and discoidal shapes, and made out of lipids and polymers, was almost totally inhibited after an overnight pre-treatment with 0.5 mM MPN. Microscopy analyses revealed that MPN reversibly reduced the extension and branching complexity of the microtubule network in BMDM, thus altering membrane bulging and motility. In immunocompetent mice, a 4 h pre-treatment with MPN was sufficient to redirect 2000 nm rigid particles from the liver to the lungs realizing a lung-to-liver accumulation ratio larger than 2. Also, in mice bearing U87-MG tumor masses, a 4 h pre-treatment with MPN enhanced the therapeutic efficacy of docetaxel-loaded nanoparticles significantly inhibiting tumor growth. The natural liver sequestering function was fully recovered overnight. This data would suggest that MPN pre-treatment could transiently and reversibly inhibit non-specific particle sequestration, thus redirecting nanomedicines towards their specific target tissue while boosting their anti-cancer efficacy and imaging capacity.

## Abstract 2

Methyl Palmitate Nanoparticles (MPN) boost the tumor accumulation and chemotherapeutic efficacy of nanomedicines by transiently and reversibly inhibiting the phagocytic properties of tissue-resident macrophages.

## Graphical abstract

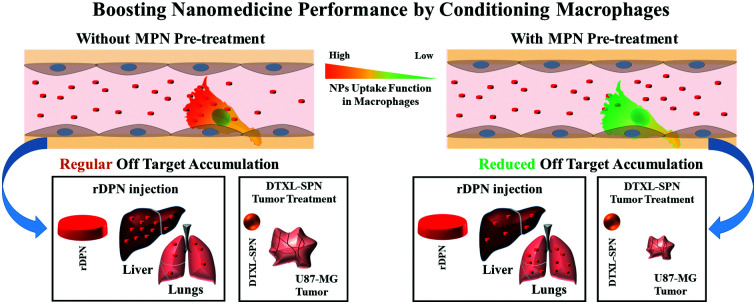

**New concepts**

The efficient and effective delivery of drug and imaging agents-loaded nanoparticles (NPs) to diseased tissues is massively limited by the non-specific sequestration in the organs of the reticulo-endothelial system. In particular, resident macrophages of the liver remove a significantly large portion of blood-borne NPs. Despite several advancements in the field, including PEGylation, tuning particle deformability, chemical removal of macrophages, the issue is far from being properly addressed, thus limiting the clinical integration of nanoparticle-based therapies. We developed a novel biological nanoparticle (MPN) that, upon systemic injection, transiently and reversibly diminishes the phagocytic capacity of resident macrophages in the liver. The systemic injection of therapeutic and imaging NPs within 4 hours post MPN administration results in enhanced diseased tissue targeting and anti-cancer efficacy. These results demonstrate the relevance of a safe modulation of NP sequestration by liver and other reticulo-endothelial organs and highlight the importance of better understanding the mechanisms by which uptake of NPs can be temporary inhibited.

## Introduction

The therapeutic and imaging capabilities of nanomedicines could be boosted by properly negotiating their interactions with multiple biological barriers, which are sequentially distributed from the site of injection up to the sub-cellular biological target.^1–3^ It is well accepted that the first and, perhaps, the major impediment to the effective delivery of therapeutic and imaging cargos *via* systemically injected nanomedicines is represented by the Mononuclear Phagocytic System (MPS).^2,4^ This collects cells of the immune system, primarily circulating monocytes and resident macrophages, whose function is to sequester and process any object that is not recognized as self. This would be harmful, xenobiotic substances; viruses; bacteria; cell debris and, indeed, also systemically administered nanomedicines.^5^ Kupffer cells, lining the walls of the liver sinusoids; splenic macrophages, distributed within the so-called white pulp of the spleen; and a vast and diverse population of tissue macrophages, distributed within the lungs, gut, brain, bone marrow and other organs, are part of the MPS.^6^ As such, a significantly large portion of blood-borne nanomedicines are estimated to deposit in the liver and spleen, regardless of their material composition and physico-chemical features.^4^ In general, different strategies have been proposed to negotiate the interaction of nanomedicines with the MPS in order to mitigate rapid sequestration and clearance from circulation. These strategies include the surface decoration of nanoparticles with polymeric chains,^7,8^ patches of cells’ membrane,^9,10^ or exosomes;^11^ tailoring the shape and deformability of nanoparticles;^12–15^ attaching nanoparticles to the surface of red blood cells;^16,17^ blockade or even depletion of macrophages from the liver and spleen.^18–22^

The surface decoration of nanoparticles with chains of polyethylene glycol (PEG) is among the most extensively used strategy. PEG chains are either covalently attached or blended on the nanoparticle surface.^7,8^ This generates a steric repulsion potential that reduces the adsorption of blood proteins (opsonins) that would otherwise facilitate the recognition by immune cells.^7,23^ This strategy is used to enhance the circulation half-life and, therefore, the tumor accumulation of liposomes and several other polymeric and inorganic nanoparticles. However, the stability of the PEG coating, as well as the continuous and growing exposure to PEG-containing dietary products, inevitably reduces the benefits of this approach.^24,25^ Another strategy is based on camouflaging nanoparticles with plasma membrane patches extracted from different cells. Erythrocyte, leucocyte, cancer cell membranes have been used, and reduced nanoparticle uptake by resident macrophages has been demonstrated *in vitro* and *in vivo*.^9–11^ However, despite promising preclinical results, this strategy involves complex procedures for membrane isolation and purification limiting its clinical translatability. More recently, tailoring the shape and deformability of nanomedicines is being proposed as an alternative strategy to mitigate recognition and internalization by professional phagocytes. First, it was documented that spherical particles are more rapidly uptaken by macrophages as compared to cylindrical and discoidal particles.^12,13^ Then, a few papers have started to demonstrate, *in vitro* and *in vivo*, that deformable particles are less easily sequestered by phagocytic cells as opposed to their rigid counterpart.^14,15^ However, only a few nanoparticle fabrication techniques allow to simultaneously control these two design parameters, which is limiting the impact of this approach. Hitchhiking nanoparticles by attaching them to long circulating red blood cells is another interesting strategy. This was originally proposed by the Mitragotri's group, whereby sufficiently small nanoparticles are transiently adsorbed over the surface of red blood cells.^16,17^ These cells are deformable, long circulating and tend to move in the core of the blood vessels, thus limiting the interaction with resident macrophages. However, this strategy often requires the *ex vivo* manipulation of red blood cells (RBC) followed by re-infusion of the RBC-nanoparticle complex. These are complex procedures that need to be performed by specialized laboratory.

The fourth strategy is based on conditioning the MPS with a specific treatment before administering the actual therapeutic or imaging nanoparticles. The objective of the treatment is to transiently block the phagocytic function of the immune cells or, in some cases, totally deplete these cells from their native organs. In the latter case, solutions like gadolinium chloride (GdCl_3_) have been administered to deplete immune cells from liver and spleen.^22^ However, this is a quite toxic and invasive intervention, given also the significant safety concerns associated with the administration of Gd^3+^-ions even with Magnetic Resonance imaging procedures.^26^ A relative less invasive approach is based on the administration of large amounts of 500 kDa Dextran Sulfate (DS) or empty liposomes. In the first case, liver blockade was observed only for doses as high as 50 mg kg^−1^ of DS, injected intraperitoneally.^20^ Note, incidentally, that DS is a highly pro-inflammatory molecule. In the second case, 400 μmol kg^−1^ of 200 nm positively charged liposomes were systemically injected to reduce by 50% the liver uptake of gold nano rods.^18^ However, there is some controversy around this latter strategy as the exposure of these particles to immune cells could also induce activation rather than inhibition.^27,28^ In view of the above observations, the identification of new compounds with an immunomodulatory, transient, and reversible effect on macrophages is highly desirable.

In this work, methyl palmitate nanoparticles (MPN) are proposed as a novel nanoparticle-based formulation to transiently and reversibly modulate the uptake function of Kupffer cells and other macrophages. MPN are composed by two endogenous compounds: the fatty acid methyl palmitate and serum albumin. In the rational design of MPN, albumin has a structural role while methyl palmitate serves to modulate the internalization capacity of macrophages. The anti-inflammatory and anti-phagocytic effect of this molecule was previously documented on several different types of macrophages, including primary Kupffer cells, Raw 267.4 cells, peritoneal macrophages and *in vivo*.^29–34^ Here, the albumin – methyl palmitate assembly into MPN is used to efficiently deliver the natural fatty acid and transiently inhibit the uptake of nanomedicines by resident macrophages. The inhibition and recover rates are quantitatively assessed *via* confocal microscopy and flow cytometry analyses. A putative biophysical mechanism underlying the effect of MPN is hypothesized by analyzing its effect on cell microtubules *via* electron, confocal fluorescent microscopy and time-lapse microscopy. Finally, the potential of MPN to affect the biodistribution of nanomedicines and improve their therapeutic outcome in cancer therapy is documented in immunocompetent mice and in U87-MG tumor bearing mice, respectively.

## Results

### Physico-chemical and biological characterizations of MPN

**Fig. 1:**
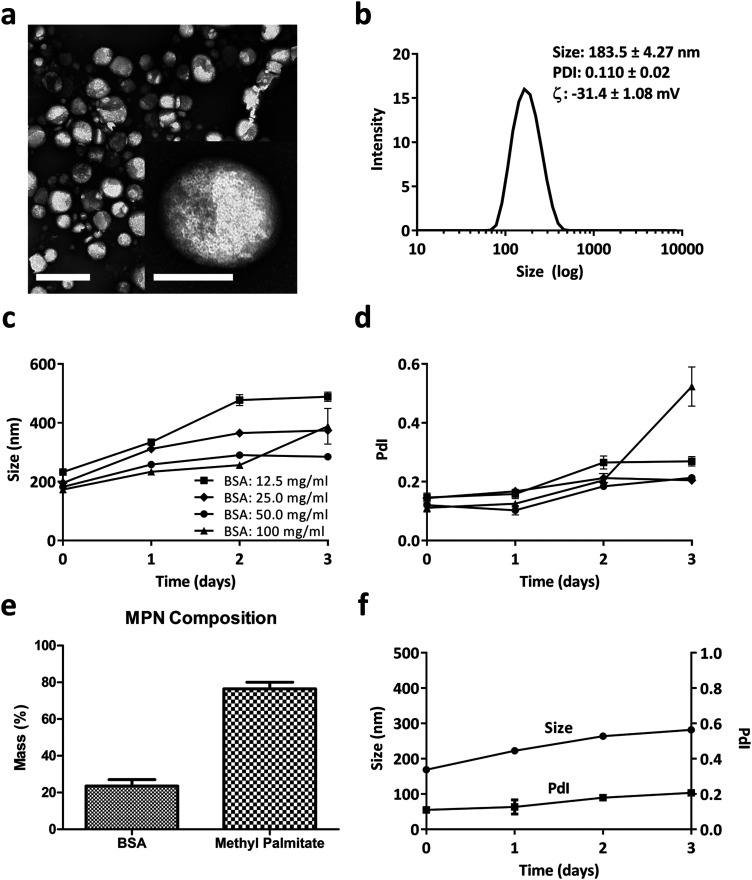
Methyl Palmitate Nanoparticles (MPN). (a) TEM image of MPN (scale bar = 0.5 μm). Inset: High magnification TEM image of a MPN (scale bar = 100 nm). (b) DLS analysis of MPN. (c and d) DLS stability analysis in PBS (hydrodynamic diameter and PdI) of MPN produced with different masses of bovine serum albumin. (e) MPN composition measured by bicinchoninic acid assay for BSA and gas chromatography-mass spectrometry for methyl palmitate. (f) DLS stability analysis in PBS (hydrodynamic diameter and PdI) of MPN realized using a 50 mg ml^−1^ BSA.

MPN were self-assembled by mixing a methyl palmitate ethanol solution to an aqueous solution of Bovine Serum Albumin (BSA) or Fetal Bovine Serum (FBS). The resulting nanoparticles appeared spherical with a quite uniform size of ∼200 nm, as per the TEM analysis of Fig. 1a. The inset at the bottom, right corner shows one single nanoparticle at high magnification. Dynamic light scattering analysis reported of nanoparticles with an average hydrodynamic diameter of 183.5 ± 4.27 nm with a narrow size distribution (PdI of 0.110 ± 0.02) and a surface electrostatic potential *ζ* of −31.4 ± 1.08 mV (Fig. 1b). In Fig. S1 (ESI†), similar data were demonstrated for MPN realized by mixing methyl palmitate ethanol solution directly with FBS. Even in this case, TEM analyses confirmed the nanoparticle sphericity while DLS reported an average hydrodynamic diameter of 172.8 ± 1.87 and a negative surface *ζ* potential of −46.9 ± 2.32 mV. Importantly, in the absence of BSA or FBS, the methyl palmitate nanoparticles did not form, as shown by the DLS data in Fig. S2 (ESI†). This image presents multiple, sparse sharp peaks that cannot be associated with any well-defined nanoparticle.

Based on this observation and given the abundance of albumin in serum, it was hypothesized that this protein is a crucial ingredient in the formation of stable and well-defined particles. Therefore, to find an ideal formulation, MPN were realized for varying BSA concentrations, ranging from 12.5 to 100 mg ml^−1^, while the methyl palmitate mass was fixed at 2 mg. As reported in Fig. 1c and d, MPN formed for all tested concentrations, namely 12.5, 25, 50, 100 mg ml^−1^, and returned an average hydrodynamic diameter of ∼200 nm and a PdI of ∼0.15. However, while the 50 mg ml^−1^ BSA formulation manifested a quite stable diameter and PdI up to 3 days of observation in PBS, all other formulations underwent a significant increase either in size or PdI, or both, over time. Consequently, the 50 mg ml^−1^ BSA formulation was selected as the ideal formulation. The actual amounts of albumin and methyl palmitate in the MPN were quantified *via* Bicinchoninic Acid Assay (BCA) and gas chromatography coupled to mass spectrometry, respectively (methods related to this second technique are presented in ESI†). Results indicated that the 50 mg ml^−1^ BSA MPN configurations contained 23.50 ± 3.55% of albumin and 76.49 ± 3.55% of methyl palmitate (Fig. 1e). Also, these MPN were quite stable for 3 days in PBS at 37 °C, as documented in Fig. 1f and Fig. S3 (ESI†) in terms of DLS hydrodynamic diameter and PdI. Similar observations also apply to the particle stability in FBS (37 °C for 24 h), as reported in Fig. S4 (ESI†). In addition, it is important to note that the MPN treatment is well tolerated by primary macrophages (Bone Marrow Derived Macrophages – BMDM), extracted from the femur of rats. No signs of toxicity emerged from MTT and propidium iodide assays and no significant variation in cytokines’ gene expression was found by real time PCR as documented in Fig. S5 and S6 (ESI†), respectively. Notably, the same safe profile was observed *in vivo* too, as shown in Fig. S7 and S8 (ESI†) in terms of hematic concentrations of the liver enzymes (AST and GPT), creatinine, and inflammatory cytokines (TNF-α, IL-6, IL-10), respectively.

### Inhibiting the internalization activity of macrophages *via* MPN

**Fig. 2:**
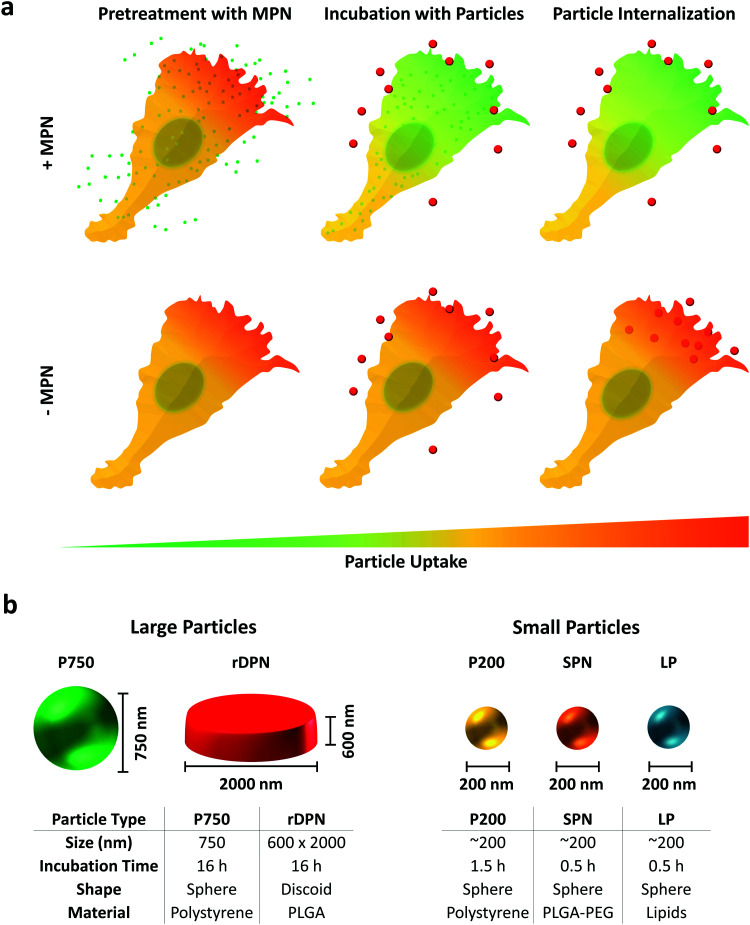
Schematic representation of the cell experiments and particles. (a) Treatment of Bone Marrow Derived Monocytes (BMDM) with MPN: cells are incubated with MPN (green dots) for 16 h; cells are incubated with large or small particles (red dots); analysis of particle internalization in BMDM following MPN pre-treatment. (b) Physico-chemical properties of large and small particles used for the internalization experiments.

BMDM were employed to characterize the inhibitory properties of methyl palmitate nanoparticles on cellular internalization processes. Experiments were performed following the schematic of Fig. 2a: BMDM were first exposed to methyl palmitate nanoparticles (small green dots) for 16 h and then exposed to different types of particles (red dots), as depicted in the top row (+MPN). In the control experiments, BMDM were not treated with MPN but the same timing for particle exposure was followed in all cases (bottom row: −MPN). For testing the internalization ability of BMDM, five particle types, with different size and shape, were considered. This is graphically shown in Fig. 2b, where also the characteristic dimensions of the five particles are listed in a table. Two types of ‘large particles’ and three types of ‘small particles’ were considered. The ‘large particles’ include the commercially available 750 nm spherical polystyrene beads (P750) and the rigid 2000 × 600 nm discoidal polymeric nanoconstructs (rDPN). The ‘small particles’ include the commercially available 200 nm polystyrene beads (P200); 200 nm spherical polymeric nanoparticles (SPN); and 200 nm liposomes (LP). The rDPN were produced by using as only component poly(lactic-*co*-glycolic acid) – PLGA while SPN were fabricated by blending together PLGA and polyethylene glycol – PEG; both particles were tagged with fluorescent RhB molecules, as previously described by the authors.^35,36^ LP were realized using dipalmitoyl-phosphatidyl-choline (DPPC), cholesterol and 1,2-distearoyl-*sn-glycero*-3-phosphoethanolamine-*N*-[amino(polyethylene glycol)-2000] (DSPE-PEG(2000)) and fabricated by thin layer evaporation, following conventional methods.^37^ The larger particles were exposed to BMDM for 16 h whereas the smaller particles were incubated with BMDM for 90 min (P200) and 30 min (SPN and LP). These different incubation times were identified in order to optimize particle uptake by BMDM. The ability of MPN to efficiently inhibit the internalization of the large and small particles was assessed *via* fluorescent confocal microscopy, using a fixed amount of MPN corresponding to 0.25 mM methyl palmitate, and *via* flow cytometry, varying the MPN concentration from 0 to 0.5 mM equivalent dose of methyl palmitate.

**Fig. 3:**
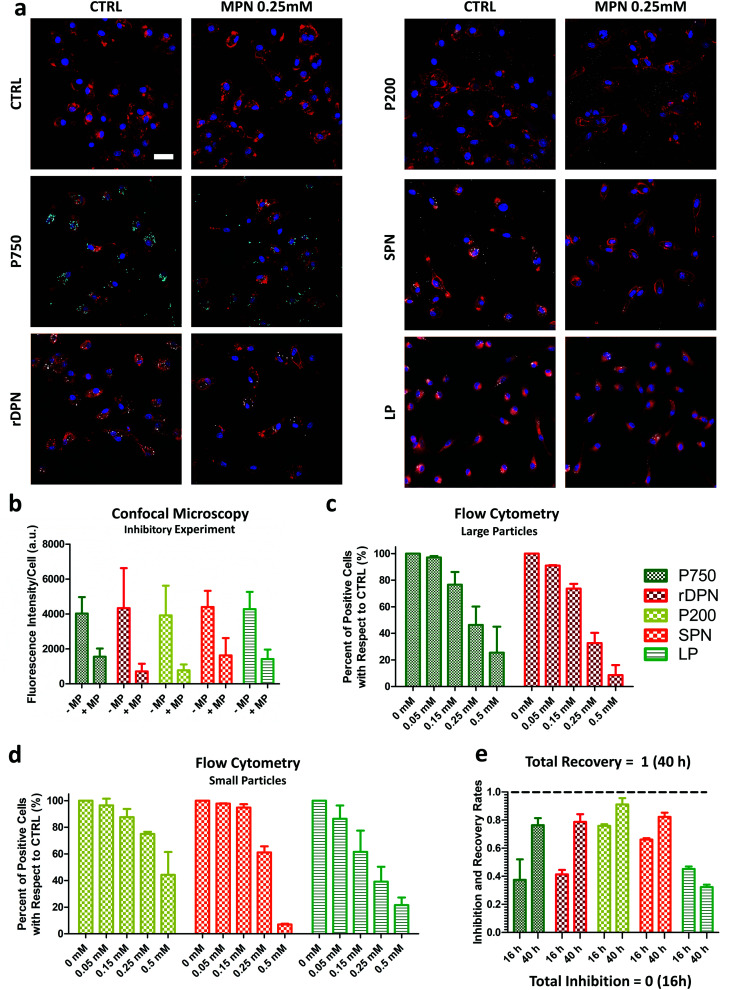
Internalization inhibition and recovery post MPN treatment. (a) Confocal microscopy maximum intensity projection images for BMDM treated with MPN and different particles. (P750 and P200: 750 nm and 200 nm) polystyrene particles; rDPN: 2 μm rigid discoidal polymeric particles; SPN: 200 nm spherical polymeric particles; and LP: 200 nm liposomes. (Membrane staining was performed by using WGA for all the condition but liposome; for liposome condition cell mask was used; nuclei were stained using DAPI.) (Scale bar = 15 μm.) (b) Confocal microscopy particle internalization quantified as fluorescence intensity/cell, measured throughout the entire cell volume. (c and d) Flow cytometry uptake experiment on BMDM treated with different amounts of MPN (dose response) and different particles (large and small particles) (e) flow cytometry inhibition/recovery assay (first column is for the inhibition rate; second column is for the recovery rate. 0 = full inhibition; 1 = full recovery). MP concentration in MPN of 0.25 mM.

Representative confocal microscopy images of BMDM exposed to the five different particles and the control experiment are provided in Fig. 3a. The cell membrane appears in red; the nuclei were stained in blue, and the particles look like well-defined white dots (P750 appears cyan blue). Z-Stacks were acquired to consider the full cell volume and identify more clearly only the fully internalized particles. The representative maximum intensity projection images of Fig. 3a document a reduced particle internalization with BMDM following a treatment with 0.25 mM MPN (right columns) as compared to the controls (left columns). Note that the polystyrene particles (P750 and P200) were observed to extensively stick to the bottom of the well, especially in the case of MPN-treated cells. The fluorescent intensity associated solely with the internalized particles is plotted in Fig. 3b, for the MPN-treated cells and control experiments. From this bar chart, it was concluded that the reduction in fluorescence intensity after the MPN treatment depends on the particle type and is generally >50%. Note that particle fluorescence intensity was measured over the entire volume of the cell, thus particles that were just associated but not internalized by the cell were not taken in account for the analysis (see Fig. S9, ESI†). It is also important to underline that since fluorescence efficiency depends on the particle type, no direct comparison was made in terms of amounts of internalized particles among the different experimental groups (*i.e.*: P750 *vs.* rDPN). (*p*-Values of these analyses are reported in the ESI.†)

For the flow cytometry analysis, large particles and small particles were again tested following the same conditions as in the confocal microscopy experiments but with varying MPN amounts, covering equivalent methyl palmitate concentrations ranging from 0 mM to 50 mM. Indeed, this was modulated by changing the MPN amounts rather than their formulation. The data in Fig. 3c and d give the percentage of positive cell with respect to 0 mM (CTRL). A cell is considered positive when associated with ‘large particles’ or ‘small particles’. The bar charts document for all five tested particles a dose dependent internalization inhibition. In other words, the number of cells associated with particles, both large and small, decreases as the amounts of MPN used in pre-treatment increases. Specifically, the percentage of positive cells for P750 reduced by about half (46.3 ± 13.8%) in 0.25 mM MPN-treated cells (Fig. 3c). Similarly, for rDPN, a MPN dose equal to 0.25 mM of methyl palmitate was sufficient to reduce the percentage of particle positive cells to 32.6 ± 7.7% of the CTRL condition. In Fig. 3d, the percent of cells positive for the uptake of P200 decreased by 74.9 ± 1.6% of the control. For SPN, the reduction was found to be equal to 61.1 ± 4.5% of the CTRL condition. For LP, the percentage of particle positive cells reduced by 39.1 ± 11.2% of the control condition. This observed response is in line with the fluorescent data presented in Fig. 3b, for the confocal microscopy analyses. An even larger inhibition on particle uptake was observed at higher MPN concentrations: 0.5 mM of methyl palmitate. Under this condition, percentage of positive cell with respect to CTRL decreased to 25.5 ± 19.5% for P750; 8.5 ± 7.6% for rDPN; 44.3 ± 17.1% for P200; 7.0 ± 0.5% for SPN and 21.5 ± 5.7% for LP (Fig. 3c and d). Note that all these experiments were performed using MPN realized with methyl palmitate and FBS (*p*-values of these analyses are reported in the ESI†). Similar results were found also with MPN obtained by mixing methyl palmitate and BSA, as shown in the Fig. S10 (ESI†). At 0.25 mM, the MPN realized with BSA were slightly more effective than those obtained with FBS for all administered particles but P750. However, no statistically significant difference was observed between the two MPN formulations at 0.5 mM.

One more fundamental question to be addresses is whether the particle uptake activity of BMDM is permanently or transiently inhibited and how rapidly it could be restored. For this reason, the flow cytometry internalization experiments were repeated considering two different times for MPN incubation. In the inhibition experiment, particles were again exposed to BMDM at 16 h post MPN-treatment; whereas in the recovery experiment, particles were exposed to BMDM at 40 h post MPN-treatment. This is graphically pictured in the Fig. S11 (ESI†). In other words, it was assumed that BMDM would recover their phagocytic function in 1 day (24 h) after the completion of the MPN treatment. A direct comparison was performed between the inhibition and recovery experiments for all five tested particles. Fig. 3e shows the rates of inhibition and recovery for the small and large particles. Note that the rate of inhibition was calculated as the ratio between the number of positive cells at 16 h post MPN treatment and the untreated cells, so that a total inhibition would correspond to a 0 inhibition rate in Fig. 3e. The rate of recovery was calculated as the ratio between the number of positive cells at 40 h post MPN treatment and the untreated cells, so that a total recovery would correspond to a 1 recovery rate in Fig. 3e. For most particles, the inhibition rates were close to 50%, in agreement with the data presented in Fig. 3c and d. This demonstrates the consistency of the data across multiple and different experiments. The recovery rates were mostly close to unity for all particle types, but the liposomes. The recovery rate was equal to 0.76 ± 0.05 for the P750; 0.79 ± 0.05 for the rDPN; 0.91 ± 0.04 for the P200; 0.82 ± 0.03 for the SPN; and only 0.32 ± 0.02 for the LP. These results would confirm the original hypothesis that in 24 h the uptake ability of the MPN-treated cells could be almost fully recovered. However, this appears not to be the case for LP, for which the cell uptake activity is instead further reduced at 40 h. The *p*-values for confocal microscopy and flow cytometry analyses are shown in the ESI.† In order to monitor the clearance time of MPN by macrophages, particles were decorated with a fluorophore. Fluorescent MPN can be easily realized by using different fluorophores (cyanine-5 (Cy5) and Rhodamine B (RhB)) as reported in Fig. S12 (ESI†). In this work, Cy5-MPN were used due to the more suitable features of the cyanine-5 molecule. As reported in Fig. S13 (ESI†), the quantitative analysis would suggest that MPN are cleared by the host cell within 24 to 48 h. This clearance could be associated with the progressive metabolization of the methyl palmitate, and albumin by the cell machinery as well as by particle exocytosis.

### Mechanisms regulating the inhibition of internalization by MPN

To gain further insights about the mechanisms regulating the inhibitory activity of MPN, multiple assays were considered, including time-lapse microscopy for assessing cell motility and electron microscopy to localize methyl palmitate nanoparticles within the cell body.

**Fig. 4:**
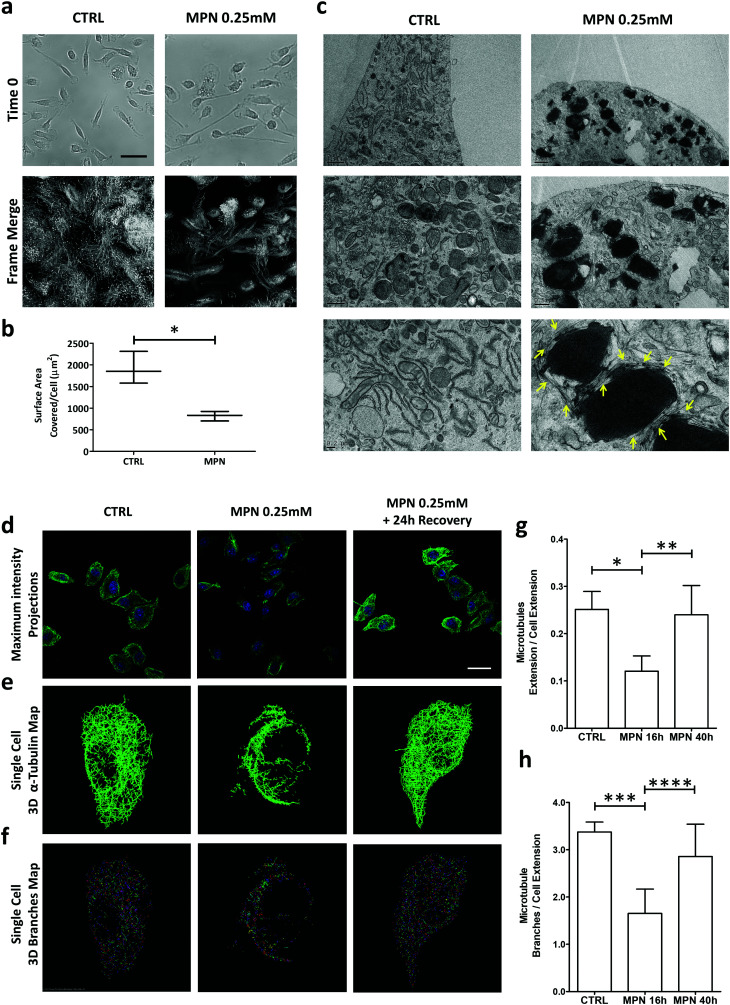
Mechanisms regulating the inhibition of internalization by MPN treatment. (a) Time lapse microscopy of BMDM treated with MPN. Top images: initial frame for both conditions. Bottom images: merged frames after 16 h of observation (scale bar = 80 μm). (b) Quantification of the surface area covered/cell over the 16 h of observation (*p = 0.008). (c) TEM images of BMDM treated with MPN (0.25 mM of MP) and untreated (CTRL). (d) Confocal microscopy maximum intensity projection for microtubule network (α-tubulin) in BMDM untreated, treated with MPN and after 24 h recover post MPN treatment (α-tubulin: green signal obtained by immunofluorescence assay; nuclei: blue signal obtained by DAPI staining) (scale bar = 20 μm). (e) Single cell 3D binary α-tubulin map. (f) Single cell 3D α tubulin branches map. (g and h) Quantification of microtubules extension and α-tubulin branches number, normalized by cell area (*p* values: * 0.0105; ** 0.0416; *** 0.0119; **** 0.1181).

Time-lapse experiments were conducted on BMDM for 16 h, corresponding to a full duration of the MPN treatment (0.25 mM of methyl palmitate). First, no visible alterations in the plasma membrane structure were detected, suggesting the absence of any detrimental effect on the cell. Then, a significant reduction in cell margin fluctuations and cell locomotion were observed for MPN-treated BMDM (Fig. 4a). To compare margins fluctuations and cell locomotion in treated and untreated cells, all the frames of 16 h-long movies were merged together into one single image highlighting the surface area physically occupied by all the cells over time. This area, normalized by the number of cells, ranged from 1915 ± 372 μm^2^ for untreated cells to 820 ± 110 μm^2^ for MPN-treated cells (*p* = 0.008) (Fig. 4b). This would indicate that the MPN treatment perturbs the ability of cells to rearrange their membranes, which is also a cellular process involved in internalization. Representative movies are available as ESI.†

Per the TEM analysis, BMDM were treated for 16 h with MPN, then fixed in 2% glutaraldehyde, post fixed with osmium tetroxide and stained overnight with uranyl acetate. Fig. 4c shows TEM micrographs of untreated (top row) and 0.25 mM MPN-treated BMDM (bottom row), respectively. In the treated cells, MPN tend to cluster forming dark spots which are surrounded by segmented tubular structures of uniform width. Under TEM measurements, these tubular structures return a diameter of 24 ± 3 nm, which is very close to the typical diameter of microtubules (∼25 nm) (Fig. S14, ESI†). The reduced capability in rearranging plasma membrane and the finding of 24 ± 3 nm ∅ tubular structures upon treatment might be related to a direct effect of MPN on microtubules, possibly destabilizing cell tensegrity. Cell shape and motility, plasma membrane bulging, phagocytosis and intracellular trafficking are typically regulated by microtubules.^38^ These are cylindrical microscopic tubes, presenting a characteristic diameter of ∼25 nm and a length varying from 200 nm to several micrometers, forming part of the cell cytoskeleton. Therefore, considering the above findings, it seems reasonable to hypothesize that the MPN-treatment could transiently affect the architecture of the microtubule network of the cell. This is schematically reported in Fig. S15 (ESI†). To directly prove that MPN alter the architecture of microtubules in treated BMDM, confocal fluorescent microscopy analyses were conducted on untreated cells (CTRL), 16 h treated cells (inhibition) and 40 h treated cells (recovery). For this experiment, cells were fixed in PFA and α-tubulin, a component of microtubules, was stained. Images were acquired in z-stack series and presented as maximum intensity projections (Fig. 4d). The image analysis provided in Fig. 4e and f highlights in 3D the extension and branching of microtubules, respectively; related movies reporting the reconstructions along the *z*-axis are available as ESI.† The analysis revealed a complex and well-organized microtubule network for the untreated cells (left column); an extensively altered and impaired microtubule architecture with a strongly diminished fluorescent for the 16 h MPN-treated cells (central column); a recovered microtubule network similar to that of the untreated cells for the 40 h MPN-treated cells (right column). Qualitatively, this data demonstrates that MPN do dramatically alter the organization of the microtubule network at 16 h post treatment and that the microtubule network recovers the original configuration at 40 h post treatment. Notably, the time frame for internalization inhibition precisely matches that of microtubule structure alteration. Similarly, the time needed to restore the microtubule homeostasis correlates with the full recovery of the uptake function.

In order to precisely estimate the differences among these three experimental conditions, the total extension and branching of the microtubules network were quantified following the procedure described in the Methods. Indeed, the larger are the extension of the microtubule network and the number of branches in the network, the more plastic is the network itself. Conversely, a limited extension of the network should be associated with a diminished capacity of the cell to rearrange its cytoskeleton and thus engulf foreign objects, as the particles. In Fig. 4g and h, the extension of the microtubule network and the branching were quantified for these three different conditions. For the 16 h MPN-treated cells, both parameters are diminished by almost 50% as compared to untreated cells and the 40 h MPN-treated cells. The difference between the CTRL (no treatment) and inhibition data, and the inhibition and recovery data is significant for both the extension (*p* = 0.0105 and 0.0416) and branching of the microtubule network (*p* = 0.0119 and 0.1181), respectively. No significant difference was documented between the untreated cells and the 40 h MPN-treated cells, demonstrating again that 24 h are sufficient to recover the original cell functions.

Taken together this data would continue to suggest that the inhibitory effect of MPN is transient and associated with a temporary reorganization of the microtubule architecture. Fig. S16–S18 (ESI†) provide additional images of BMDM treated with MPN for 16 h and 40 h, confirming again the microtubule network alterations observed.

### Modulating particle biodistribution *via* MPN

Following the data presented in the previous paragraphs it could be speculated that the systemic administration of MPN into animals could induce a temporarily lethargy of macrophages residing in organs of the reticulo-endothelial system. The spherical shape and the hydrodynamic diameter (200 nm) together favor MPN uptake by resident macrophages in the liver, which are indeed the direct biological target of MPN. In order to support this statement, a biodistribution study was performed using the *in vivo* imaging system (IVIS) in C57BL/6J mice (Charles River, USA). This revealed a high liver uptake of MPN within the first 2 h post-injection, as documented in Fig. S19 (ESI†). Cy5-MPN were used for this experiment (please see Fig. S12, ESI†). Once particles are uptaken, Kupffer cells should have a diminished capacity to sequester blood-borne particles, which, instead, would circulate longer and accumulate more at their target site.

In the present paragraph and in the following one, two different types of particles were considered as test-cases: a micrometric, rigid particle that would tend naturally to accumulate in the liver and in the lungs; a 200 mm particle that could reach a tumor exploiting the enhanced permeability and retention effect and deploy its anti-cancer therapeutic cargo (docetaxel) thereof.

**Fig. 5:**
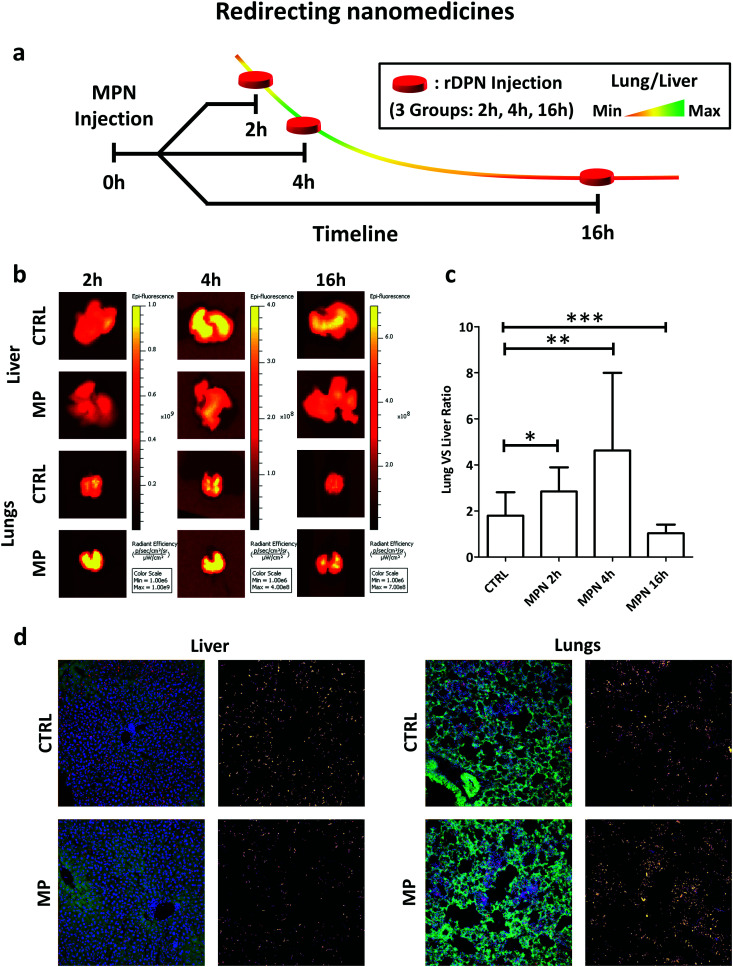
*In vivo* performance of nanomedicines post MPN treatment. (a) Schematic representation of preclinical experiments demonstrating the redirection of Cy5-rDPN from liver to lungs in immunocompetent mice pre-treated with MPN (mass of methyl palmitate: 3.75 ± 0.73 mg); (b) *ex vivo* IVIS imaging of lung and liver tissues harvested from immunocompetent mice (red signal is proportional to rDPN tissue deposition). (c) Quantification of rDPN lung/liver deposition at 2, 4 and 6 h post MPN administration. CTRL is for untreated mice (*p*-values: * 0.0426; ** 0.0044; *** 0.0935). (d) Representative confocal images of histological sections of liver and lungs of healthy mice which injected with 1 billion of rDPN in presence or absence of MPN pretreatment (green = autofluorescence from cells; blue = nuclei (DAPI); red = particles).

Among all tested particles, the 2000 × 600 nm rigid discoidal polymeric nanoconstructs (rDPN) were specifically designed to be easily recognized and uptaken by macrophages.^14^ Also, given the size and rigidity, rDPN would manifest a specific tropism for the pulmonary microvasculature.^39–41^ As such, the biodistribution of rDPN in C57BL/6 (Charles River – USA) immunocompetent mice (*n* ≥ 5) was assessed with and without MPN treatment in liver and lungs. A schematic representation of the experimental plan in shown in Fig. 5a. 3 batches of MPN were administered per animal by tail vein injection (this dose is equivalent to a methyl palmitate mass of 3.75 ± 0.73 mg). 1 billion of Cy5-labelled rDPN were injected systemically at 2, 4 and 16 h post MPN administration as well as in mice lacking the MPN treatment (CTRL). The rDPN accumulation into the liver and lungs was quantified using an IVIS system 2 h post injection. The fluorescence associated with rDPN in the hepatic and pulmonary tissues is qualitatively shown in Fig. 5b, whereas the radiance efficiency ratio between lungs and liver is plotted in Fig. 5c. In control animals, this ratio is higher than unity (1.79 ± 1.02), indicating a larger rDPN accumulation in the lungs *vs.* the liver. Differently, in treated mice, this ratio is equal to 2.85 ± 1.05 already at 2 h post MPN administration. Then, it grows to reach a maximum of 4.63 ± 3.37 at 4 h and reduces back again to the control value (*p* = 0.0935) at 16 h. This data demonstrates that 2 h and 4 h pre-treatments with MPN are sufficient to redirect rDPN (*p* = 0.0426 and *p* = 0.0044 compared to control) towards the lungs, while the phagocytic function of the Kupffer cells is recovered after 16 h. Given that the function of these macrophages was restored quite rapidly, it can be assumed that MPN do not induced any permanent effect on the immune system cells. Note that for both the inhibitory and recovery experiments, shorter characteristic times were sufficient *in vivo* as compared to the *in vitro* experiments. The transient inhibition of particle internalization following the MPN administration would allow to redirect systemically injected nanomedicines from the reticulo-endothelial organs to the biological target. Representative images of histological section of the lungs and liver are shown in Fig. 5d. The same condition of the *in vivo* imaging experiments with the IVIS were replicated, but only the 4 h MPN pretreatment time was considered. In the images, nuclei were stained by DAPI, cell bodies are shown in green and particle in red. Next to each image, the Cy5 channel is reported (Lookup table: fire from ImageJ Software was used to produce this images) to highlight the different accumulation of the particles with and without MPN pretreatment.

### Improving the anticancer efficacy of nanomedicines *via* MPN

**Fig. 6:**
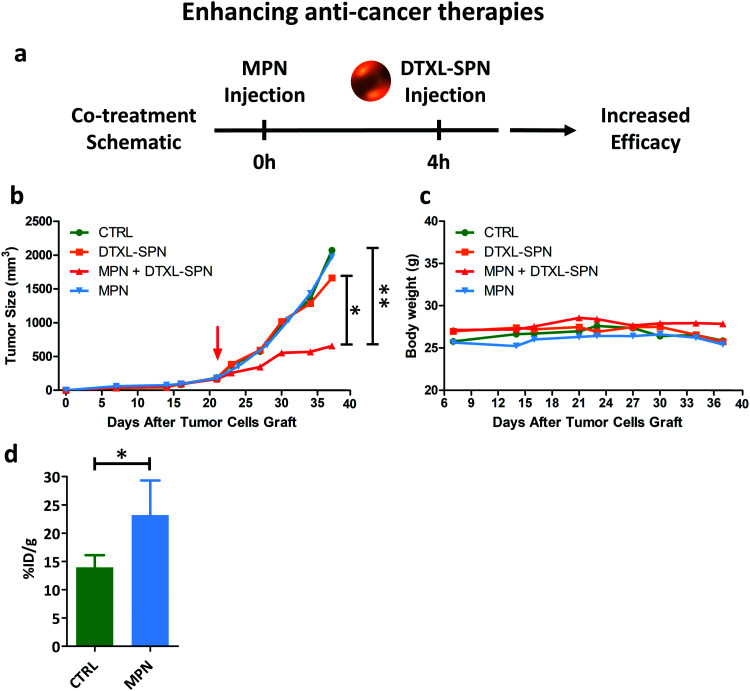
*In vivo* performance of nanomedicines post MPN treatment II. (a) Schematic representation of the co-treatment (DTXL-SPN – 4.5 mg kg^−1^ of DTXL every 3.5 days) in U87-MG bearing mice pre-treated with MPN (mass of methyl palmitate: 3.75 ± 0.73 mg); (b) variation of the tumor volume over time. Red arrow indicates treatment initiation. Results are presented as mean (*n* ≥ 3) (*p* values: * 0.051; ** = 0.014); (c) variation in mouse body weight during treatment. (d) Increased tumor accumulation of ^64^Cu-SPN in U87-MG bearing mice pre-treated with MPN (mass of methyl palmitate: 3.75 ± 0.73 mg) *p value = 0.0354 (*n* ≥ 3).

While in the previous paragraph, MPN pre-treatment was found to reduce the notorious non-specific hepatic deposition of nanomedicines, in this section, the enhancement in therapeutic performance of docetaxel-loaded nanomedicines is investigated. 200 nm spherical polymeric nanoparticles loaded with docetaxel (DTXL-SPN) (Fig. S20, ESI†) were selected to treat a malignant mass originated in the flank of 7 weeks old nude mice (Charles River, USA) upon the subcutaneous injection of U87-MG cells. An equivalent dose of 4.5 mg kg^−1^ of DTXL was systemically administered twice a week for a total of 6 treatments. A schematic representation of the co-treatment is shown in Fig. 6a.

The variation of the tumor volume over time was monitored and is documented in Fig. 6b. Mice treated with saline and DTXL-SPN had both their malignant masses rapidly growing over ∼1000 mm^3^ after already 30 days post inoculation. Note that, the DTXL-SPN alone were injected following a different treatment plan as compared to previous studies of the authors^42^ in order to highlight the efficacy of the MPN pre-treatment. More specifically, in the present work, DTXL-SPN were administered twice a week at an equivalent DTXL dose of 4.5 mg kg^−1^ as compared to one injection every other day at 3.0 mg kg^−1^ DTXL. Thus, under the considered treatment schedule, the sole administration of DTXL-SPN was not sufficient to arrest tumor growth, whereas the administration of DTXL-SPN at 4 h post MPN treatment significantly inhibit tumor growth (Fig. 6b – *n* ≥ 3, see ESI,† for all *p*-values). The comparison in tumor growth between MPN treated and untreated mice indicates that the methylpalmitate nanoparticles *per se* would not exert any anti-tumoral activity, at least in the current experimental configuration. No significant changes in body weight were reported, as shown in Fig. 6c, suggesting that no toxicity is associated with the multiple treatments. With the objective of demonstrating that the enhanced antitumor efficacy of DTXL-SPN is directly associated with their higher deposition within the tumor tissue, U87-MG carrying mice were injected with ^64^Cu-SPN, in the presence or absence of MPN pretreatment. The same treatment scheme adopted for the therapeutic experiments was considered. At 24 h post ^64^Cu-SPN administration, mice were euthanized, their major organs were collected and measured for radioactivity *via* a scintillation γ-counter. This analysis documented a 60% increase in SPN accumulation within the tumor tissue following a MPN pre-treatment (Fig. 6d). The synthesis protocol and the characterization of ^64^Cu-SPN are available in Methods and in Fig. S21 (ESI†). A complete ^64^Cu-SPN biodistribution analysis was conducted and included in the Fig. S22 (ESI†) for U87-MG tumor bearing mice. Importantly, more than 80% of the injected ^64^Cu dose was recovered at 24 h within the analyzed organs, demonstrating the stable chelation of the radioisotope to the SPN.

## Discussion

The potential of nanomedicines to deploy large doses of active therapeutic agents within the diseased tissues, enabling truly combinatorial therapies and proper patient stratification *via* minimally invasive imaging, is hampered by the mononuclear phagocytic system. Indeed, it is well documented that nanomedicines can reach malignant tissues at concentrations as high as 20% of the injected dose per mass of tissue.^4^ These values are generally several orders of magnitude higher than those associated with more conventional chemotherapeutic small molecules. Still, off-target organs are hit at significantly high doses, regardless of the nanoparticle nature, such as the liver and the spleen. These filtering organs are populated by millions of resident macrophages whose function is to sequester out of the blood circulation any not-self object. Minimizing non-specific accumulation within the liver and the spleen would surely boost the circulation half-life and deposition of nanomedicines at their pre-determined biological target. In this work, this objective was achieved by designing methyl palmitate nanoparticles (MPN) for inducing transiently a state of dormancy in Kupffer cells, and possibly other macrophages.

Methyl palmitate is a hydrophobic fatty acid that has been known for its anti-inflammatory and anti-phagocytic properties for a long time.^29–32,34^ The reformulation of this molecule into stable 200 nm nanoparticles (MPN) upon mixing with serum albumin represents a simple strategy to deliver methyl palmitate to immune cells. Interestingly, these MPN are stable only for a specific range of mass ratios between methyl palmitate and albumin. Larger or smaller methyl palmitate-to-albumin ratios would not conduce to stable MPN formulations, thus limiting the ability to efficiently deliver methyl palmitate to macrophages. After characterizing the physico-chemical properties of MPN, their inhibitory activity on internalization by primary macrophages was demonstrated considering particles ranging in size from 200 up to 2000 nm, exhibiting a spherical and a discoidal shape, and being made out of lipids and polymers. In all cases, a nearly 50% inhibition in uptake was registered at 16 h post-treatment with MPN. This was documented using two different experimental techniques, as confocal fluorescent microscopy and flow cytometry. More importantly, a recovery of about 90% of the cell internalization ability was observed only 24 h post MPN treatment completion. This clearly demonstrated that the effect of MPN is reversible and transient. As the different internalization mechanisms are all associated with cell membrane rearrangement and bulging, electron and confocal fluorescent microscopies were extensively used to monitor the transformation of the microtubule network in primary macrophages upon treatment with MPN. Electron microscopy images would suggest that MPN can perturb microtubules network, perhaps destabilizing it. Indeed, fluorescent microscopy images of α-tubulin stained cells showed a microtubule network with a shorter extension and smaller number of branches following MPN administration. This could be related to the diminished internalization activity. Indeed, 24 h post MPN treatment completion, the structure of the microtubule network was observed to be similar to the original, untreated configuration. All this data pointed towards the role of MPN in reversibly and transiently alter the organization of the microtubule network.

Moreover, the methyl palmitate nanoparticles were tested in immunocompetent mice to demonstrate that a pre-administration with MPN could diminish accumulation within the liver and boost deposition at the pre-determined target organ. For this experiment, 2000 × 600 nm rigid discoidal polymeric nanoconstructs (rDPN) were used in that these particles would extensively accumulate in the liver and lungs. Indeed, in the control experiments, rDPN were observed also in the liver, with a lung-to-liver ratio of about 2. However, this accumulation was reduced in favor of the lungs at 2 and 4 h post MPN administration. This continues to demonstrate that methyl palmitate included in the MPN, reaches the Kupffer cells in the liver, and most likely other macrophages too, and in already 2 h is capable to relax the phagocytic activity of these cells. The liver accumulation is even more inhibited at 4 h post MPN administration, whereby the rDPN lung-to-liver accumulation ratio becomes larger than 2. Even *in vivo*, the inhibition is transient and after only 16 h the initial conditions are recovered again. Notably, the timing for inhibition and recovery *in vivo* is faster than *in vitro*, suggesting that the proposed inhibition strategy could be considered even less invasive in animals as compare to cell cultures.

Finally, to prove that the reduced liver accumulation of nanoparticles could enhance the therapeutic efficacy of nanomedicines, tumor bearing mice were first pre-treated with MPN and then administered with a suspension of anti-cancer nanomedicines. Following the biodistribution data, DTXL-SPN were administered systemically at 4 h post MPN treatment. While DTXL-SPN alone failed to control tumor growth, the MPN pre-treatment was sufficient to redirect DTXL-SPN towards the malignant mass and, consequently, boost the anti-tumor efficacy of nanomedicines.

## Conclusions

The natural fatty acid methyl palmitate was reformulated into stable 200 nm nanoparticles upon mixing with albumin, or serum. The resulting nanoparticles were employed to transiently induce a dormant state into primary macrophages thus inhibiting their internalization capacity. This inhibition reduced by about 50% the uptake of nanomedicines, over a broad range of sizes, shapes, and compositions. Total recovery of the internalization capacity was also documented, indicating that the inhibition process is reversible and transient. This was demonstrated *in vitro* on primary bone marrow derived monocytes and *in vivo* in immune competent mice. The pre-administration of MPN was shown to change the fate of systemically injected nanomedicines boosting their specific tissue accumulation and therapeutic performance.

## Materials and methods

### Fabrication and characterization of MPN

MPN were produced by using a self-assembly method: a solution 1 : 1 (V : V) of methyl palmitate and ethanol containing 2 mg of methyl palmitate was added to 100 μl of a 50 mg ml^−1^ BSA solution (in some of the analysis 100 μl of FBS were used instead). The obtained mixture was sonicated for 1′ into a water bath, washed in 1 ml of D.I. water and centrifuged at 15 000 rpm at 4 °C. Particles were washed and finally re-suspended in 1 ml of PBS or water (depending on the specific need) and sonicated for 1′. To understand the ideal concentration of BSA solution used for the MPN assembly, MPN were also complexed by using BSA solutions ranging from 12.5 mg ml^−1^ to 100 mg ml^−1^. Transmission electron microscopy (TEM) micrographs were acquired using JEOL JEM 1011 (Jeol, Japan) electron microscope (Electron Microscopy Facility, Fondazione Istituto Italiano di Tecnologia, Genoa – Italy) operating with an acceleration voltage of 100 kV and recorded with a 11 Mp fiber optical charge-coupled device (CCD) camera (Gatan Orius SC-1000). MPN samples were diluted 1 : 100, dropped on 150-mesh glow discharged ‘ultrathin’ carbon-coated Copper TEM grids, dried and directly observed. Average size, size distribution, and zeta potential of MPN were analyzed using dynamic light scattering. Samples were diluted with isosmotic double distilled water (1 : 100, v/v) to avoid multiscattering phenomena and analyzed at 25 °C with a Zetasizer Nano (Malvern, UK), equipped with a 4.5 mW laser diode and operating at 670 nm as a light source, and the scattered photons were detected at 173°. A third order cumulative fitting autocorrelation function is applied to measure the average size and size distributions. The analysis was carried out according to the following instrumental setup: (a) a real refractive index of 1.59; (b) an imaginary refractive index of 0.0; (c) a medium refractive index of 1.330; (d) a medium viscosity of 1.0 mPa s; and (e) a medium dielectric constant of 80.4. The actual amounts of serum albumin and methyl palmitate in the MPN were quantified *via* bicinchoninic acid assay kit (Euro Clone, Italy) and gas chromatography coupled to mass spectrometry, respectively. Bicinchoninic Acid Assay was used according to vendor indications. For stability analysis both in PBS and FBS, particles were kept at 37 °C under agitation for the whole period of analysis, small aliquots (5 μl) of the samples were taken at the different time point and diluted in 1 ml of D.I. water in order to run DLS analyses.

### Fabrication and characterization of the small and large particles

Rigid discoidal polymeric nanoconstructs (rDPN) were synthesized by employing a top-down fabrication process. Briefly, this involves the use of the direct laser writer lithographic technique to fabricate a silicon master template presenting an array of discoidal holes with circular base and fixed size. This pattern is then replicated into PDMS (Sylgard® 184, Dow Corniging, USA) and subsequently poly(vinyl alcohol) (PVA) (Sigma Aldrich, USA) templates, by using soft lithography. Once the holes of the sacrificial template (PVA) are filled with the polymeric mixture, composed by the hydrophobic (poly(lactic-*co*-glycolic acid)) (PLGA) (Sigma Aldrich, USA) and lipid Cy5, the PVA is dissolved in water to collect the resulting particles. For the *in vitro* experiments, lipid Rhodamine-B (Avanti Polar Lipids, USA) is added to the polymeric paste composing the DPN instead of lipid Cy5. Multisizer Coulter counter (Beckman Coulter, USA) was used to count particles.

Spherical polymeric nanoparticles (SPN) were synthesized by employing an emulsion/solvent evaporation technique. DSPE-PEG was dissolved in a 4% ethanol solution to a final volume of 3 ml to obtain the aqueous phase, whereas 1 mg of PLGA and an appropriate quantity of DPPC and of PE lissamine rhodamine B were dissolved in chloroform to create the oil phase. A v/v ratio of 6 : 1 between the aqueous and organic phase, a lipids/polymer w/w ratio of 20% and a DPPC/DSPE-PEG molar ratio of 7.5 : 2.5 were used. Then, the oil phase was added in a dropwise manner to the aqueous solution under ultrasonication at 60% amplitude (Q125 Sonicator, Q-Sonica). The resulting emulsion was then gently stirred at room temperature and in a reduced pressure environment for 4 hours to allow solvent evaporation. Finally, nanoparticles were centrifuged at 1500 rpm for 5′ to remove any possible debris obtained in the synthesis process, surnatant was again centrifuged at 12 700 rpm for 20′, pellet underwent 3 washes with water. For the *in vivo* experiment, DTXL-SPN were similarly prepared. DTXL, in a 10 : 1 ratio with PLGA, was included in the oil phase and the same procedure described above was followed. More details of this specific preparation are available elsewhere.^42^

Liposomes were prepared by thin layer evaporation (TLE). Briefly DPPC, cholesterol, DSPE-PEG and DSPE-CY5 were dissolved in chloroform in a round bottomed flask (ratio 6 : 3 : 1 : 1). The thin layer lipid film was obtained with the evaporation of the organic solvent at 60° under reduce pressure. The lipid film was left under the hood overnight to remove any trace of residual solvent. For the production of the multilamellar liposomes, the lipid film was hydrated with 2 ml of HEPES and then to three alternate cycles (3 min each) of warming at 60 °C (thermostated water bath) and vortexing at 700 rpm. The sample was dialyses against HEPES for 1 h.

### Bone marrow derived monocytes harvesting and culturing

Bone marrow derived monocytes (BMDMs) from rats were isolated based on the following procedures. Briefly after scarifying the animal, femurs were isolated, cleaned from surrounding tissues and washed in PBS (Thermo Fisher Scientific, USA), a cut was performed at both ends. PBS was used to flush the cavities, cells were harvested and plated in media supplemented with macrophage Colony-Stimulating Factor (mCSF) (10 ng ml^−1^) (Sigma Aldrich, USA). Culturing medium was changed after 3 days to remove unattached cells. BMDMs were used on the following day. Culture media: DMEM supplemented with 15% FBS, 1% penicillin/streptomycin, and rat M-CSF (Sigma Aldrich, USA) (according to vendor indications). Cells were cultured under controlled environmental conditions (37 °C in 5% CO_2_).

### Particle internalization analysis *via* confocal microscopy

For cells imaging 65.000 BMDMs were seeded into each well of a Nunc™ Lab-Tek™ II Chamber Slide™ System (Thermo Fisher Scientific, USA) maintaining culturing conditions, as described above. Cells were treated overnight with MPN (16 h) and with particles of different size and material according to their internalization timing. For treatment with big particles: 10 particles per cell were used for P750 and rDPN. For treatment with small particles: 100 particles per cell were used for P200, 25 μl of SPN suspension were used (corresponding to a total mass of ∼30 μg and to a 20 μg ml^−1^ final concentration) and 10 μl of liposomes suspension were used (corresponding to a total mass of ∼20 μg and to a 13.3 μg ml^−1^ final concentration). P750 and 2 μm rDPN were added to the cells right after MPN treatment; P200 and SPN and liposomes were added to the cells 16 h after MPN treatment (treatment lasted 90′ and 30′ and 30′ respectively). After these treatments media was removed and cells were washed in PBS (Thermo Fisher Scientific, USA). Fixation was performed using a 3.7% solution of paraformaldehyde (Sigma Aldrich, USA) for 10 minutes; 3 washes were performed after cell fixation. For the internalization inhibition analysis, plasma membrane was stained using Alexa Fluor™ 647 WGA (Thermo Fisher Scientific, USA) according to vendor's protocol. In the panel in which the internalization of liposomes is investigated CellMask™ (Thermo Fisher Scientific, USA) was used instead of WGA. A 63× objective was used and a z-stack series was acquired (≥12 steps of 1000 nm each were acquired per image). Images were realized by using a A1-Nikon confocal microscope (Nikon Corporation, Japan).

### Particle internalization analysis *via* flow cytometry

Flow cytometry was performed using a FACS ARIA (Becton Dickinson, USA). 200.000 BMDMs were seeded into each well of a 12 well plate maintaining culturing conditions indicated in cell culturing section. Cells were treated according to the same indication described for Confocal Microscopy. After treatment cells were washed in DMEM, high glucose, no glutamine, no phenol red (Thermo Fisher Scientific, USA) and harvested in 200 μl of the same media using a scraper to detach cells from the plastic support. Samples were stored in ice and vortexed right before the analysis. For the analysis of the restored internalization capability, after the overnight treatment with MPN the media was removed and replaced with medium without MPN (after performing 2 washes with PBS). Same downstream procedures were applied.

### Data analysis

Cell population was selected setting a scatter gate excluding the negligible amount of debris and aggregates present in the samples and taking in account the side scatter shift due to internal complexity changes caused by internalized particles. The population of cell positive for internalization was selected considering the basal level of fluorescence in untreated cells.

### Time lapse experiment

For time-lapse microscopy experiments, BMDMs were seeded into a Nunc™ Lab-Tek™ II Chamber Slide™ System (Thermo Fisher Scientific, USA) maintaining culturing conditions, as described above. A Nikon Eclipse-Ti-E microscope (Nikon Corporation, Japan) was used for this analysis. 8 h after cell seeding MPN were added and time lapse movies were acquired in bright-field for 16 h. Movies were acquired at a frame rate of 0.2 fpm using a 20× objective. 3 fields per condition were acquired, and statistical analyses were performed considering the total space occupied by cells over time. Statistical analyses were performed using ANOVA. *P*-Value was equal to 0.008. Data are presented as means ± SD.

### Transmission electron microscopy analysis of BMDM

Transmission electron microscopy (TEM) micrographs were acquired using JEOL JEM 1011 (Jeol, Japan) electron microscope (Electron Microscopy Facility, Fondazione Istituto Italiano di Tecnologia, Genoa – Italy) operating with an acceleration voltage of 100 kV and recorded with a 11 Mp fiber optical charge-coupled device (CCD) camera (Gatan Orius SC-1000). 500.000 BMDMs were seeded into each well of a 6 wells plate. A sterilized borosilicate glass was previously placed into each well; culturing conditions were maintained as described above. Cells were treated overnight with MPN, samples were fixed for 2 h in 1.5% glutaraldehyde in 0.1 M sodium cacodylate buffer (pH 7.4), post fixed in 1% osmium tetroxide in the same buffer and stained overnight with 1% uranyl acetate aqueous solution. Samples were then dehydrated in a graded ethanol series, infiltrated with series of ethanol/resin solution and finally embedded in epoxy resin (Epon 812, TAAB). Thin sections were cut with Leica UC6 ultra-microtome (Leica Microsystems, Germany) equipped with a diamond knife (Diatome).

### α-Tubulin immunofluorescence and microtubules analysis

For cells imaging 65.000 BMDMs were seeded into each well of a Nunc™ Lab-Tek™ II Chamber Slide™ System (Thermo Fisher Scientific, USA) maintaining culturing conditions, as described above. After these treatments media was removed and cells were washed in PBS (Thermo Fisher Scientific, USA). Fixation was performed using a 3.7% solution of paraformaldehyde (Sigma Aldrich, USA) for 10 minutes; 3 washes were performed after cell fixation. Images of microtubules architecture were obtained by immunostaining; α-tubulin antibody (Sigma – T5168) was used as primary antibody and Abcam – ab6786 was used as secondary antibody according to vendors indications. A 100× objective was used and a z-stack series was performed (≥21 steps of 300 nm each were acquired per image). Images were acquired by using an A1-Nikon confocal microscope (Nikon Corporation, Japan). For both analyses, nuclei were stained using DAPI (Thermo Fisher Scientific, USA) following vendors indications. For the analysis of the recovery of microtubules structure and the restored internalization capability, the media was removed after the overnight treatment with MPN, 1 wash with PBS was performer and medium was replaced.

For α-tubulin analysis, the following procedure was followed: (i) for each of the images composing a z-stack series, a binary 2D map of α-tubulin was generated (sample images are provided in Fig. S16–S18, ESI†). (ii) 2D binary maps from the same z-stack series were used to generate a binary 3D α-tubulin map (Fig. 4d). (iii) Binary 3D α-tubulin map were used to find α-tubulin branches (Fig. 4e) (movies report the z-stack reconstruction of the cells, ESI†). Following these analyses, it was possible to quantify the total microtubules extension and the number of microtubules branches inside each of the cells and for the 3 different conditions (both measures were normalized over cell extension) (*n* ≥ 10). For the generation of 2D α-tubulin map same threshold was selected for all the focal planes, the binary image was processed to define the microtubules using NIS-Software (Nikon Corporation, Japan). The normalization over cell extension was performed by selecting a permissive threshold (highlighting the whole cell body) on tubulin signal on a maximum intensity projection image generated by each of the z-stack series. Normalization on cell extension was preferred over normalization on cell number due to the difference in cell extension among cells. Statistical analyses were performed using ANOVA. Data are presented as means ± SD.

### Cy5-rDPN biodistribution experiment

7 weeks old female C57BL/6J mice (Charles River) were feed with special diet for 1 week to reduce the fluorescence derived by food. MPN were intravenously administered, the amount of MP used is equal to 3.75 mg per animal. Animals were treated for 2 h, 4 h and 16 h and subsequently injected with 2 μm Cy5-rDPN, control group was only treated with 2 μm Cy5-rDPN. 2 h after the second injection animals were sacrificed, liver and lungs were observed using IVIS (PerkinElmer, USA). Anesthesia was performed using isoflurane by inhalation. Statistical analyses were performed using ANOVA. Data are presented as means ± SD. In a separate session, C57BL/6J mice (Charles River – USA) were treated according to the same protocol, only 4 h MPN pretreatment was considered for this experiment. 2 h after rDPN injection, animals were sacrificed; liver and lungs were harvested and placed into a falcon tube containing PBS PFA 4%. Samples were stored at 4 °C for 2 days, than washed in PBS 3 times. PBS was than replaced with a 40% (w/v) PBS Sucrose solution and samples were stored at 4 °C for 2 more days. Samples were than included into OCT (Leica, Wetzlar, Germany), frozen in dry ice and stored at −80 °C. Samples were then cut in 20 μm slices and deposited on histology slides (VWR, Radnor, PA, USA). Samples were than stained by using a 1 : 1000 DAPI solution (Thermo Fisher Scientific, USA) for 1 h. A coverglass was mounted by the addition of ProLong™ Gold Antifade Mountant (Thermo Fisher Scientific, USA) and samples were observed by confocal microscopy by using a 20× Nikon objective (a z-stack series was performed of ≥21 steps of 1000 nm each were acquired per image) mounted on a A1-Nikon confocal microscope (Nikon Corporation, Japan).

### U87-MG treatment *in vivo*

U87-MG cells were cultured in T-175 flasks (Corning, USA) under controlled environmental conditions (37 °C in 5% CO2). EMEM, supplemented with 15% FBS, 1% penicillin/streptomycin (Sigma Aldrich, USA), was used as culturing medium. Cells were harvested on the day of inoculation in female CD-1 nude mice (7 weeks old) purchased from Charles River Laboratories. U87-MG cells were inoculated in the flank by subcutaneously injecting a 1 : 1 suspension of Matrigel (Corning, USA)/PBS containing 1 million of cells. Tumors with a volume of ∼180 mm^3^ were considered for starting the therapy ∼21 days post cell inoculation. Mice were randomly selected and assigned to the 4 different experimental groups: saline (CTRL), DTXL-SPN, MPN + DTXL-SPN and MPN alone. Both formulations (MPN and DTXL-SPN) were intravascularly administered. More specifically: in DTXL-SPN and MPN + DTXL-SPN groups DTXL-SPN were administered retro-orbitally; in MPN + DTXL-SPN and MPN alone groups, a tail vein injection was performed for the administration of MPN, 4 hours prior to DTXL-SPN administration. Treatments were performed bi-weekly for a total of 6 administrations, mice were sacrificed 37 days post cell inoculation. An equivalent dose of 4.5 mg kg^−1^ DTXL was used for the treatments. Tumor growth (caliper measure) and body weight were also monitored bi-weekly. Anesthesia was performed with isoflurane for retro-orbital injection and for tumor measuring. The following formula was applied to calculate tumor volume: 1/2(length × width^2^).

### Tumor accumulation of ^64^Cu-SPN

U87-MG tumor bearing nude mice (Charles River, USA) were injected with ^64^Cu-SPN in order to measure particles accumulation inside tumor, in presence or absence of MPN pretreatment following the same treatment scheme operated in the therapy experiment. 24 h upon the injection with ^64^Cu-SPN the mice were euthanized, tumors were collected and weighed. The radioactivity was measured with a scintillation γ-counter (WIZARD 2480, PerkinElmer, Waltham, MA).

### Ethics oversight

All animal experiments were performed according to the guidelines established by the European Communities Council Directive (Directive 2010/63/EU of 22 September 2010) and approved by the National Council on Animal Care of the Italian Ministry of Health (License: 883/2017 *et al.*).

## Author contributions

R. P.: designed and prepared MPN; performed internalization experiments by confocal microscopy and flow cytometry; performed time-lapse microscopy experiment; performed α-tubulin analysis; performed *in vivo* experiments; wrote the paper. M. D. F.: performed DLS analyses on all MPN formulations; prepared LP and DTXL-SPN. V. D. F.: prepared SPN, LP and DTXL-SPN. F. P.: provided technical assistance for all the *in vivo* experiments. T. C.: performed TEM analysis on BMDM. M. F.: prepared DSPE-Cy5 and ^64^Cu-SPN and performed the ^64^Cu-SPN *in vivo* experiment. A. L. P.: prepared rDPN for *in vitro* and *in vivo* studies and performed the ^64^Cu-SPN *in vivo* experiment. P. D.: contributed to the development of MPN and supervised the overall study; wrote the paper.

## Conflicts of interest

There are no conflicts to declare.

## Supplementary Material

MH-008-D1MH00937K-s001

MH-008-D1MH00937K-s002

MH-008-D1MH00937K-s003

MH-008-D1MH00937K-s004

MH-008-D1MH00937K-s005

MH-008-D1MH00937K-s006

MH-008-D1MH00937K-s007

MH-008-D1MH00937K-s008

MH-008-D1MH00937K-s009

## References

[cit1] Jain R. K., Stylianopoulos T. (2010). Delivering nanomedicine to solid tumors. Nat. Rev. Clin. Oncol..

[cit2] Anchordoquy T. J., Barenholz Y., Boraschi D., Chorny M., Decuzzi P., Dobrovolskaia M. A., Farhangrazi Z. S., Farrell D., Gabizon A. and Ghandehari H., Mechanisms and barriers in cancer nanomedicine: addressing challenges, looking for solutions. ACS Publications, 2017 10.1021/acsnano.6b08244 PMC5542883 28068099

[cit3] Moghimi S. M., Hunter A. C., Murray J. C. (2005). Nanomedicine: current status and future prospects. FASEB J..

[cit4] Wilhelm S., Tavares A. J., Dai Q., Ohta S., Audet J., Dvorak H. F., Chan W. C. (2016). Analysis of nanoparticle delivery to tumours. Nat. Rev. Mater..

[cit5] Boraschi D., Italiani P., Palomba R., Decuzzi P., Duschl A., Fadeel B. and Moghimi S. M., Nanoparticles and innate immunity: new perspectives on host defence, Seminars in immunology, Elsevier, 2017, pp. 33–51 10.1016/j.smim.2017.08.013 28869063

[cit6] Davies L. C., Jenkins S. J., Allen J. E., Taylor P. R. (2013). Tissue-resident macrophages. Nat. Immunol..

[cit7] Owens III D. E., Peppas N. A. (2006). Opsonization, biodistribution, and pharmacokinetics of polymeric nanoparticles. Int. J. Pharm..

[cit8] Perry J. L., Reuter K. G., Kai M. P., Herlihy K. P., Jones S. W., Luft J. C., Napier M., Bear J. E., DeSimone J. M. (2012). PEGylated PRINT nanoparticles: the impact of PEG density on protein binding, macrophage association, biodistribution, and pharmacokinetics. Nano Lett..

[cit9] Hu C.-M. J., Zhang L., Aryal S., Cheung C., Fang R. H., Zhang L. (2011). Erythrocyte membrane-camouflaged polymeric nanoparticles as a biomimetic delivery platform. Proc. Natl. Acad. Sci. U. S. A..

[cit10] Parodi A., Quattrocchi N., Van De Ven A. L., Chiappini C., Evangelopoulos M., Martinez J. O., Brown B. S., Khaled S. Z., Yazdi I. K., Enzo M. V. (2013). Synthetic nanoparticles functionalized with biomimetic leukocyte membranes possess cell-like functions. Nat. Nanotechnol..

[cit11] Yong T., Zhang X., Bie N., Zhang H., Zhang X., Li F., Hakeem A., Hu J., Gan L., Santos H. A. (2019). Tumor exosome-based nanoparticles are efficient drug carriers for chemotherapy. Nat. Commun..

[cit12] Champion J. A., Mitragotri S. (2006). Role of target geometry in phagocytosis. Proc. Natl. Acad. Sci. U. S. A..

[cit13] Decuzzi P., Pasqualini R., Arap W., Ferrari M. (2009). Intravascular delivery of particulate systems: does geometry really matter?. Pharm. Res..

[cit14] Palomba R., Palange A. L., Rizzuti I. F., Ferreira M., Cervadoro A., Barbato M. G., Canale C., Decuzzi P. (2018). Modulating phagocytic cell sequestration by tailoring nanoconstruct softness. ACS Nano.

[cit15] Anselmo A. C., Zhang M., Kumar S., Vogus D. R., Menegatti S., Helgeson M. E., Mitragotri S. (2015). Elasticity of nanoparticles influences their blood circulation, phagocytosis, endocytosis, and targeting. ACS Nano.

[cit16] Anselmo A. C., Gupta V., Zern B. J., Pan D., Zakrewsky M., Muzykantov V., Mitragotri S. (2013). Delivering nanoparticles to lungs while avoiding liver and spleen through adsorption on red blood cells. ACS Nano.

[cit17] Muzykantov V. R. (2010). Drug delivery by red blood cells: vascular carriers designed by mother nature. Expert Opin. Drug Delivery.

[cit18] Sun X., Yan X., Jacobson O., Sun W., Wang Z., Tong X., Xia Y., Ling D., Chen X. (2017). Improved tumor uptake by optimizing liposome based RES blockade strategy. Theranostics.

[cit19] Liu T., Choi H., Zhou R., Chen I.-W. (2015). RES blockade: A strategy for boosting efficiency of nanoparticle drug. Nano Today.

[cit20] Patel K. R., Li M. P., Baldeschwieler J. D. (1983). Suppression of liver uptake of liposomes by dextran sulfate 500. Proc. Natl. Acad. Sci. U. S. A..

[cit21] Van Rooijen N., Van Nieuwmegen R. (1984). Elimination of phagocytic cells in the spleen after intravenous injection of liposome-encapsulated dichloromethylene diphosphonate. Cell Tissue Res..

[cit22] Diagaradjane P., Deorukhkar A., Gelovani J. G., Maru D. M., Krishnan S. (2010). Gadolinium chloride augments tumor-specific imaging of targeted quantum dots *in vivo*. ACS Nano.

[cit23] Pannuzzo M., Esposito S., Wu L.-P., Key J., Aryal S., Celia C., di Marzio L., Moghimi S. M., Decuzzi P. (2020). Overcoming Nanoparticle-Mediated Complement Activation by Surface PEG-Pairing. Nano Lett..

[cit24] Suk J. S., Xu Q., Kim N., Hanes J., Ensign L. M. (2016). PEGylation as a strategy for improving nanoparticle-based drug and gene delivery. Adv. Drug Delivery Rev..

[cit25] Parr M. J., Ansell S. M., Choi L. S., Cullis P. R. (1994). Factors influencing the retention and chemical stability of poly(ethylene glycol)-lipid conjugates incorporated into large unilamellar vesicles. Biochim. Biophys. Acta, Biomembr..

[cit26] Sadowski E. A., Bennett L. K., Chan M. R., Wentland A. L., Garrett A. L., Garrett R. W., Djamali A. (2007). Nephrogenic systemic fibrosis: risk factors and incidence estimation. Radiology.

[cit27] Pervin M., Golbar H. M., Bondoc A., Izawa T., Kuwamura M., Yamate J. (2016). Transient effects of empty liposomes on hepatic macrophage populations in rats. J. Toxicol. Pathol..

[cit28] Moghimi S. M., Hunter A. C. (2001). Recognition by macrophages and liver cells of opsonized phospholipid vesicles and phospholipid headgroups. Pharm. Res..

[cit29] Cai P., Kaphalia B., Ansari G. (2005). Methyl palmitate: inhibitor of phagocytosis in primary rat Kupffer cells. Toxicology.

[cit30] El-Demerdash E. (2011). Anti-inflammatory and antifibrotic effects of methyl palmitate. Toxicol. Appl. Pharmacol..

[cit31] Marzi I., Cowper K., Takei Y., Lindert K., Lemasters J. J., Thurman R. G. (1991). Methyl palmitate prevents Kupffer cell activation and improves survival after orthotopic liver transplantation in the rat. Transplant Int..

[cit32] Sarkar S., Khan M. F., Kaphalia B. S., Ansari G. (2006). Methyl palmitate inhibits lipopolysaccharide-stimulated phagocytic activity of rat peritoneal macrophages. J. Biochem. Mol. Toxicol..

[cit33] Wooles W. t., Luzio Di, Reticuloendothelial N. (1963). function and the immune response. Science.

[cit34] Rodolfo Paoletti D. K., Advances in Lipid Research, Academic Press, New York and London, 1972, vol. 10, pp. 1–385

[cit35] Key J., Palange A. L., Gentile F., Aryal S., Stigliano C., Di Mascolo D., De Rosa E., Cho M., Lee Y., Singh J. (2015). Soft discoidal polymeric nanoconstructs resist macrophage uptake and enhance vascular targeting in tumors. ACS Nano.

[cit36] Di Francesco M., Primavera R., Romanelli D., Palomba R., Pereira R. C., Catelani T., Celia C., Di Marzio L., Fresta M., Di Mascolo D. (2018). Hierarchical Microplates as Drug Depots with Controlled Geometry, Rigidity, and Therapeutic Efficacy. ACS Appl. Mater. Interfaces.

[cit37] Szoka F., Papahadjopoulos D. (1978). Procedure for preparation of liposomes with large internal aqueous space and high capture by reverse-phase evaporation. Proc. Natl. Acad. Sci. U. S. A..

[cit38] Cooper G. M. and Hausman R. E., The cell: a molecular approach, 2004

[cit39] Muro S., Garnacho C., Champion J. A., Leferovich J., Gajewski C., Schuchman E. H., Mitragotri S., Muzykantov V. R. (2008). Control of endothelial targeting and intracellular delivery of therapeutic enzymes by modulating the size and shape of ICAM-1-targeted carriers. Mol. Ther..

[cit40] Kolhar P., Anselmo A. C., Gupta V., Pant K., Prabhakarpandian B., Ruoslahti E., Mitragotri S. (2013). Using shape effects to target antibody-coated nanoparticles to lung and brain endothelium. Proc. Natl. Acad. Sci. U. S. A..

[cit41] Xu R., Zhang G., Mai J., Deng X., Segura-Ibarra V., Wu S., Shen J., Liu H., Hu Z., Chen L. (2016). An injectable nanoparticle generator enhances delivery of cancer therapeutics. Nat. Biotechnol..

[cit42] Lee A., Di Mascolo D., Francardi M., Piccardi F., Bandiera T., Decuzzi P. (2016). Spherical polymeric nanoconstructs for combined chemotherapeutic and anti-inflammatory therapies. Nanomedicine.

