# Supplementary material for: Boosting nanomedicine performance by conditioning macrophages with methyl palmitate nanoparticles
Source: Mater Horiz. 2021 Jul 29;8(10):2726–41. doi: 10.1039/d1mh00937k (PMC8489400; doi:10.1039/d1mh00937k)
Supplement: MH-008-D1MH00937K-s005 [file MH-008-D1MH00937K-s005.pdf]

Electronic Supplementary Material (ESI) for Materials Horizons.
This journal is © The Royal Society of Chemistry 2021

BOOSTING NANOMEDICINE PERFORMANCE BY CONDITIONING MACROPHAGES WITH METHYL PALMITATE NANOPARTICLES

Roberto Palomba[1], Martina di Francesco[1], Valentina di Francesco[1,2], Federica Piccardi[3], Tiziano Catelani[4], Miguel Ferreira[1], Anna Lisa Palange[1], Paolo Decuzzi[1]

[1] Laboratory of Nanotechnology for Precision Medicine – Fondazione Istituto Italiano di Tecnologia, Via Morego 30, 16163, Genova.

[2]Department of Informatics, Bioengineering, Robotics and System Engineering, University of Genoa, Via Opera Pia, 13 Genoa 16145, Italy.

[3] Animal Facility - Fondazione Istituto Italiano di Tecnologia, Via Morego 30, 16163, Genova.

[4] Electron Microscopy Facility - Fondazione Istituto Italiano di Tecnologia, Via Morego 30, 16163, Genova.

Corresponding author: Paolo Decuzzi Ph.D., paolo.decuzzi@iit.it

SUPPLEMENTARY MATERIALS AND METHODS

Gas chromatography coupled to mass spectrometry for MP quantification in MPN. Methyl palmitate quantification was performed using a GC-ion trap MS system. The instrument consists of a Trace GC Ultra gas chromatograph equipped with an AI1310 autosampler and coupled with an ITQ 1100 ion-trap mass spectrometer (Thermo Fisher Scientific Inc., Austin, Texas, USA). The chromatographic analysis was performed on a TraceGOLD TG-5MS (5% phenyl, 95% methylpolysiloxane) fused-silica capillary column (30m x 0.25 mm x 0.25 um). The oven temperature program was 150°C (held for 4min) to 270°C at 10°C min-1 and to 310°C at 40°C min-1 (held for 4min). Helium was used as carrier gas at a constant flow-rate of 1 ml min-1. The injector temperature was kept at 250°C and samples were injected in the splitless injection mode (1 min). The transfer line and ion source temperatures were kept at 280 and 250° C respectively. The electron energy and emission current were 70 eV and 250 uA. Parameters such as automatic gain control and multiplier were set by automatic tuning. Precursor ion selected in MS-MS mode was 227 m/z and product ions were in the range 80-227 m/z. The trapping voltages were adjusted setting as automatic the collision energy. For methyl palmitate extraction from MPN 75ul of isopropanol were added to 75ul one MPN batch, the suspension was vortexed for 1 min and 500ul of Heptane were added twice to avoid losing material. Samples were than vortexed again for 1 min and samples were centrifuged at 14800 rpm for 10 minutes. The organic phases were collected and evaporated to dryness under nitrogen. Reconstitution was done with 500uL of Heptane right before injection.

MTT and propidium iodide assay. MTT assay was performed on BMDM cells after treating them with MPN (**Supplementary Figure 5a**). MPN were used in different equivalent

concentrations of methyl palmitate (0; 0.031; 0.062; 0.125; 0.250; 0.50; 1 mM). 20,000 BMDM were seeded into each well of a 96 well plate (Corning, USA). Cells were cultured according to the same condition indicated in the manuscript. 24h after cell seeding, cells were treated with MPN for 24h. At the end of the treatment MTT Formazan (1-(4,5-Dimethylthiazol-2-yl)-3,5-diphenylformazan, Thiazolyl blue formazan) (Merk-Sigma, USA) was added to the culture according vendor's indication. Cells were incubated for 4h with the reagent and the plate was than analyzed following vendor's protocol. Propidium Iodide assay (**Supplementary Figure 5b**) was instead performed following similar indication. 200,000 BMDM were seeded into a 12 multi-wheel plate (Corning, USA) 24h before the treatment. MPN treatment was performed at a concentration of methyl palmitate equal to 0.25 mM. The treatment lasted 24h. Cells were than washed in PBS twice and gently detached from the bottom of the plate by using a scraper in a final volume of 200 µl and transferred into an Eppendorf tube (Eppendorf, Germany). Propidium iodide (Merk–Sigma, USA) was added to the cells according to vendor's indication and the sample was quickly analyzed by flow cytometry.

Real Time PCR. 400,000 BMDM were seeded into a 6 well plate (Corning, USA) 24h before the treatment. MPN treatment was performed at a concentration of methyl palmitate equal to 0.25 mM. The treatment lasted 24h. Cells were than washed in PBS twice and the procedure for isolating RNA started by lysing the cells inside the plate. RNA was extracted using a RNAeasy Plus Mini Kit (Qiagen, Germany) and quantified by NanoDrop2000 (Thermo Scientific, Waltham, USA). Real-Time (RT-PCR) is used to measure mRNA levels of inflammatory cytokines. For each condition, four biological replicates were run in triplicate. RT-PCR reactions were carried out using a Power SYBR Green RNA-to-CT 1-Step Kit (Applied Biosystems, Waltham, USA) and

using GAPDH expression as a housekeeping gene reference. Reactions were performed in a final volume of 20 μL. Oligonucleotide primer pairs are as follows: for GAPDH, 5′-GAACATCATCCCTGCATCCA-3′ and 5 ′-CCAGTGAGCTTCCCGTTCA-3′; for TNF-α,5′-GGTGCCTATGTCTCAGCCTCTT-3′ and 5′-GCCATAGAACTGATGAGAGGGAG-3′; for IL-1β, 5′-TGGACCTTCCAGGATGAGGACA-3′ and 5′ GTTCATCTCGGAGCCTGTAGTG-3′; for IL-6, 5′-TACCACTTCACAAGTCGGAGGC-3′ and 5′-CTGCAAGTGCATCATCGTTGTTC-3′.

Serum transaminase, creatinine and cytokines levels. The serum concentration of aspartate aminotransferase (AST or GOT), alanine aminotransferase (ALT or GPT) creatinine, Tumor Necrosis Factor-α(TNF-α),Interleukin-6 (IL-6) and Interleukin-10 (IL-10) were measured in vivo. These measurements were performed on healthy, 8 weeks old C57BL/6J mice (Charles River, USA), before and after treating them with two systemic administration of MPN in a week. Blood was collected 1 week before the treatment and 1 day after the second treatment. ELISA tests were performed on the sera of 12 animals pulled together according to vendor instruction. The following kits were used for these analyses: Aspartate Aminotransferase (AST) (Mouse) ELISA Kit (E4320-100) (Biovision, Milpitas, USA); Alanine Aminotransferase 1 (GPT) (Mouse) ELISA Kit (E4324-100) (Biovision, Milpitas, USA); Creatinine (Mouse) ELISA Kit (E4369-100) (Biovision, Milpitas, USA); TNF alpha Mouse Instant ELISA™ Kit (BMS607-2INST) (Thermo Fisher Scientific, Waltham, USA); IL-6 Mouse ELISA Kit (KMC0061) (Thermo Fisher Scientific, Waltham, USA); IL-10 Mouse Instant ELISA™ Kit (BMS614INST) (Thermo Fisher Scientific, Waltham, USA).

3D Deconvolution of a Single Cell z-stack: One single cell z-stack was deconvolved and reconstructed in 3D by using NIS-Software (Nikon) (**Supplementary Figure 9**). For this experiment the same condition indicated in the section particle internalization analysis via Confocal Microscopy were followed.

MPN-BSA, MPN-FBA Formulation Comparison. The comparison between MPN generated by using BSA or FBS was made analyzing by flow cytometry BMDM which were treated with the two formulations and with particles (P750, rDPN, P200, SPN, LP); same indication of other flow cytometry experiments were applied in terms of particle amounts and treatment time. The internalization inhibition % was calculated according to the following formula: 100 – inhibition rate %; for FBS formulation data were derived from experimental data available in Figure 3. For BSA formulation data were derived from n=3 independent experiments (**Supplementary Figure 10**).

Fluorescent-MPN (Cy5-MPN and RhB-MPN) synthesis and characterization. Cy5-MPN were synthetized following the same procedures followed for unstained MPN. The 1:1 (V:V) solution of methyl palmitate and ethanol containing 2 mg of methyl palmitate was enriched with 700 μg/ml of DSPE-Cy5 and added to 100 μl of a 50 mg/ml BSA solution. Size distribution, PDI and ζ-potential were measured by DLS, following the same procedure indicated in the text for the unstained MPN (**Supplementary Figure 12a**). RhB-MPN were synthetized following a procedure similar to the one above described for Cy5-MPN. Lissamine-Rhodamine B (Avanti Polar Lipids, USA) was used instead of DSPE-Cy5 (**Supplementary Figure 12b**). The release profile of Cy5 from MPN was obtained by dialysis in 4 liters of PBS (infinity sink condition) (**Supplementary**

Figure 12c). The formulation was loaded inside a Slide-A-Lyzer MINI dialysis micro-tubes with a molecular cutoff of 10 kDa (Thermo Scientific, Waltam, USA) and dialyzed against 4 L of PBS buffer. For each time point, three samples (200 µl each) were collected and dried. Samples were then dissolved in acetonitrile, and analyzed by spectrophotometer.

MPN clearance from BMDM. In order to determine the permanence of MPN inside BMDM, cells were treated with Cy5-MPN at different time points (1h, 2h, 4h, 24h, 48h, 72h). For the characterization of Cy5-MPN please see the dedicated paragraph below. The analysis was run by using two different techniques: flow cytometry and confocal microscopy (**Supplementary Figure 13**). Flow cytometry was performed using a FACS ARIA (Becton Dickinson, USA). 200.000 BMDMs were seeded into each well of a 12 well plate maintaining culturing conditions indicated in cell culturing section (main text). After the cells adhered to the bottom of the wells Cy5-MPN were added (different dosing were used for this experiment, namely: 0.062mM, 0.125mM, 0.25mM). After treatment, cells were washed in DMEM (high glucose, no glutamine, no phenol red) (Thermo Fisher Scientific, Waltam, USA) and harvested in 200 µl of the same media using a scraper to detach cells from the plastic support. Samples were stored in ice and vortexed right before the analysis. *Data Analysis.* Cell population was selected setting a scatter gate excluding the negligible amount of debris and aggregates present in the samples and taking in account the side scatter shift due to internal complexity changes caused by internalized particles. The population of cell positive for internalization was selected considering the basal level of fluorescence in untreated cells.

Confocal Microscopy was performed by seeding 65.000 BMDMs into each well of a Nunc™ Lab-Tek™ II Chamber Slide™ System (Thermo Fisher Scientific, Waltam, USA) maintaining

culturing conditions, as described in in cell culturing section (main text). Cells were treated with 0.25mM of MPN for the same time points indicated for flow cytometry analysis. After treatments, media was removed and cells were washed in PBS (Thermo Fisher Scientific, Waltam, USA). Fixation was performed using a 3.7% solution of paraformaldehyde (Sigma Aldrich, USA) for 10 minutes; 3 washes were performed after cell fixation. A 63X objective was used and a z-stack series was acquired (≥12 steps of 1,000 nm each were acquired per image). Images were realized by using a A1-Nikon confocal microscope (Nikon Corporation, Japan).

3D α-tubulin Map and Tubulin Branches. Slices of one sample cell per experimental point (**Supplementary Figure 16-18**) were derived from the 3D reconstruction of α-tubulin map image and α-tubulin branches image shown in **Figure 4c-e** by using NIS-Software (Nikon Corporation, Japan). The supplementary movies show the z-stack reconstruction of the α-tubulin extension and branches binary images.

MPN Biodistribution. MPN biodistribution was analyzed by using a IVIS Spectrum In Vivo Imaging System (Perkin Elmer - Waltham, USA) (**Supplementary Figure 19**). 12 8 weeks old female C57BL/6J mice (Charles River, USA) were feed with special diet for 1 week to reduce the fluorescence derived by food. Cy5-MPN were intravenously administered (mass of methyl palmitate: ~1,87 mg/20g). Animals were animals were sacrificed after 2h, 4h and 6h and 24h. Liver, spleen, heart, lungs, brain, kidneys were isolated and images were acquired. Anesthesia was performed using isoflurane by inhalation and all the procedure were conducted following the guidelines of the Institutional Animal Care and Use Committee of IIT.

Synthesis of Docetaxel loaded SPN (DTXL-SPN). DTXL-SPN were synthesized using an oil-in water emulsion/solvent evaporation technique, as previously reported by the authors. Briefly, an organic phase (Chloroform) containing lactic-co-glycolic acid (PLGA, 10 mg), 1,2-dipalmitoyl-sn-glycero-3-phosphocholine (DPPC, 0.9 mg) and Docetaxel (DTXL, 2 mg) (Alfa Easer, Massachusetts, USA) was added to an aqueous phase (4% EtOH, 10mL) containing 1,2-distearoyl-sn-glycero-3-phosphoethanolamine-N-[carboxy(polyethylene glycol)-2000] (DSPE-PEG, 1.1 mg) and sonicated using a probe sonicator (1.30 min at 100% amplitude, Q125 sonicator, Q-Sonica). The obtained emulsion was maintained under magnetically stirring for 3h for promoting completed solvent evaporation. Particles were collected and purified by differential centrifugation steps. Size distribution of DTXL-SPN was measured at DLS together with PDI and ζ-potential, following the same indication used for MPN in the main text and reported in **Supplementary Figure 20a**.

Evaluation of Docetaxel release profile from DTXL-SPN. In order to mimic the sink conditions of blood circulation system, the release profile of DTXL from DTXL- SPN were conducted in 4L of PBS buffer (1X, pH = 7.4, 37 ± 2 °C). Specifically, 200 μL DTXL- SPN were placed into Slide-A-Lyzer MINI dialysis micro tubes with a molecular cutoff of 10 kDa (Thermo Scientific, Waltam, USA) and then dialyzed against 4 L of PBS buffer (1X, pH = 7.4, 37 ± 2 °C). At each time point, three samples were collected and centrifuged. The obtained pellet was disrupted using Acetonitrile (AcN) / H2O in a ratio 1:1, v/v. The portion of Docetaxel remaining entrapped in SPN was quantified using High Performance Liquid Chromatography (HPLC) analysis. An isocratic condition (H2O + 0.1% (v/v) TFA / AcN + 0.1% (v/v) TFA, 43:57 v/v, 0.3 mL/min) was used for its detection. DTXL was analyzed at λ of 230 nm. Results are reported in **Supplementary Figure 20b.**

Synthesis and characterization of Docetaxel loaded ^{64}Cu-SPN. For the ^{64}Cu-SPN intratumoral accumulation quantification, DOTA-SPN were labelled with ^{64}Cu. For the preparation of empty DOTA-SPN the same protocol above described for DTXL-SPN was used with some slight modification. DTXL was not included in the organic phase and DSPE-DOTA was added in the aqueous phase, in a quantity replacing 20% of DSPE-PEG. For ^{64}Cu labelling, DOTA-SPN were left to react with 500 µCi for 2h in acetate buffer at 37°C. ^{64}Cu excess was removed from the solution through 4 centrifugation cycles (2 in PBS and 2 in water, 15 min at 12.400 rpm). Size distribution of DOTA-SPN was measured at DLS together with PDI, following the same indication used for MPN in the main text (prior to ^{64}Cu binding) (**Supplementary Figure 21a**). The release profile of ^{64}Cu from SPN was determined by following the same indication provided above for DTXL release profile form SPN. The portion of ^{64}Cu remaining entrapped in SPN was quantified by gamma counter and reported in **Supplementary Figure 21b.**

^{64}Cu-SPN Biodistribution Experiment. U87-MG tumor bearing nude mice (Charles River, USA) were injected with ^{64}Cu-SPN in order to measure particles accumulation inside tumor, in presence or absence of MPN pretreatment following the same treatment scheme operated in the therapy experiment. 24h upon the injection with ^{64}Cu-SPN the mice were euthanized, organs were collected and weighed. The radioactivity was measured with a scintillation γ-counter (WIZARD 2480, PerkinElmer, Waltham, MA).

SUPPLEMENTARY RESULTS

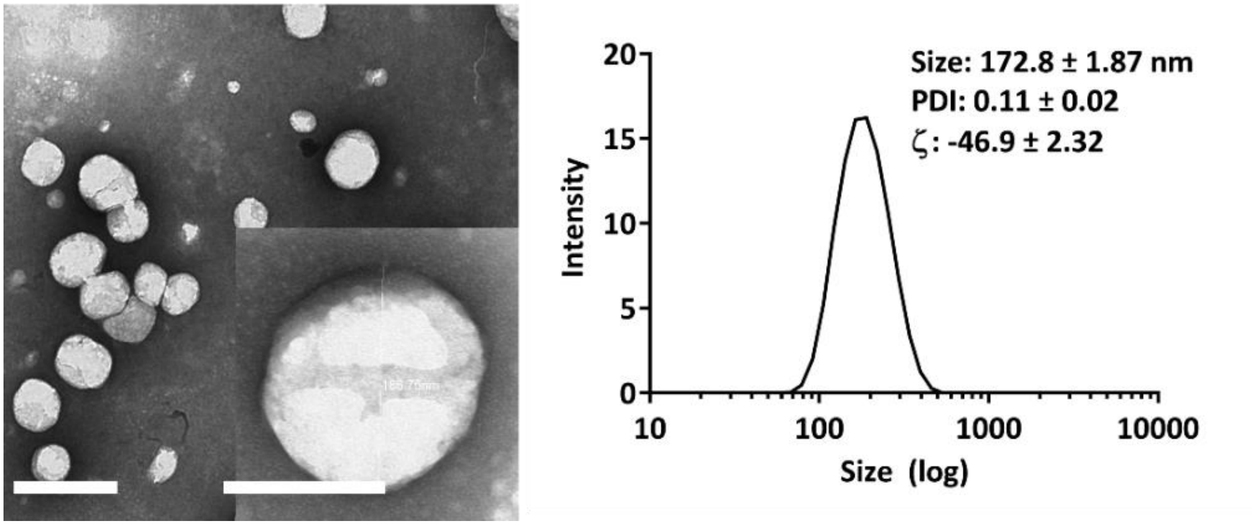

Supplementary Figure 1. TEM images and DLS analysis of MPN produced by using FBS (scale bar= 0.5 µm and 0.1 µm respectively).

Sample	Size (nm)	PdI
0 mg BSA	8875 ± 9810	0.97 ± 0.08

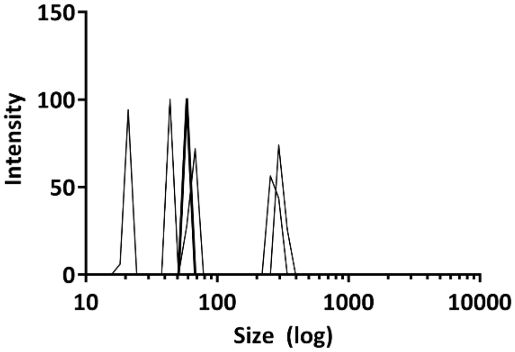

Supplementary Figure 2. DLS analysis of a no BSA or FBS formulation. (N=3)

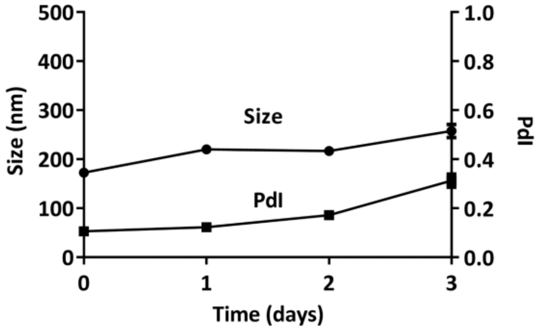

Supplementary Figure 3. Stability analysis in PBS at 37°C of MPN produced by using FBS (DLS). (N=3)

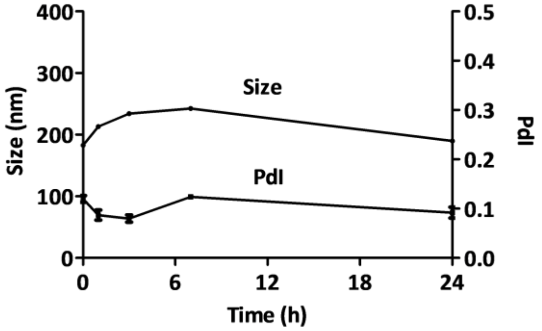

Supplementary Figure 4. Stability analysis of the MPN in FBS, at 37°C, via Dynamic Light Scattering returning the hydrodynamic diameter and Polydispersity Index (PDI) over time (n = 3)

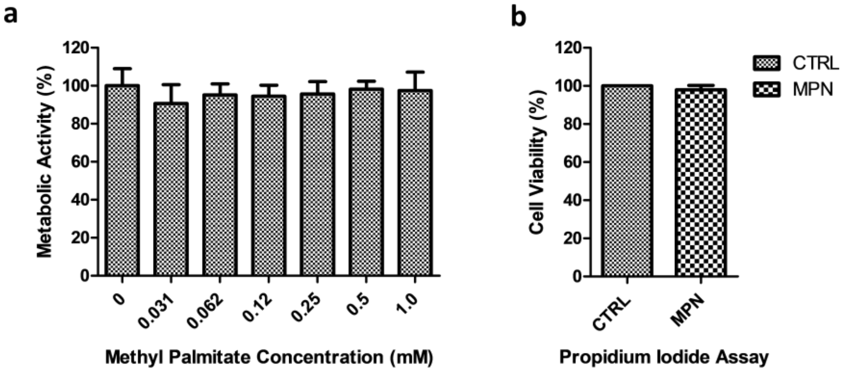

Supplementary Figure 5. In vitro toxicity analyses a. MTT assay b. propidium iodide assay on primary rat macrophages (Bone Marrow Derived Monocytes).

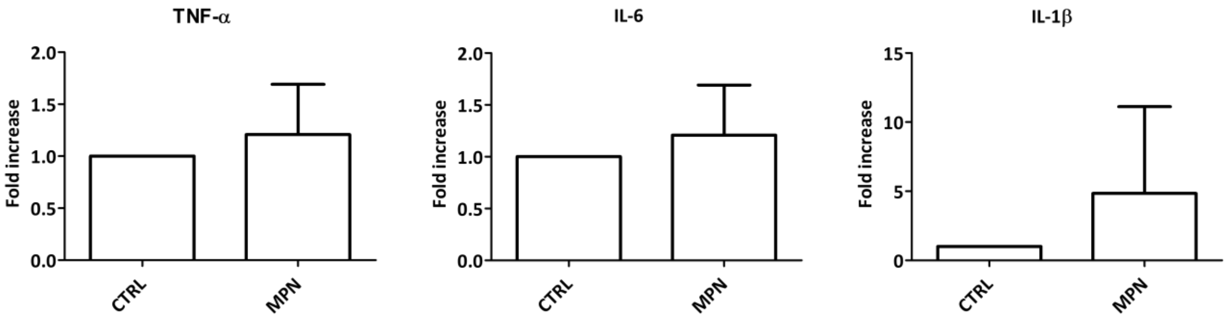

Supplementary Figure 6. Tumor Necrosis Factor-α (TNF-α), Interleukin-6 (IL-6), Interleukin-1β gene expression in BMDM treated or not with MPN. Real Time PCR (n=4).

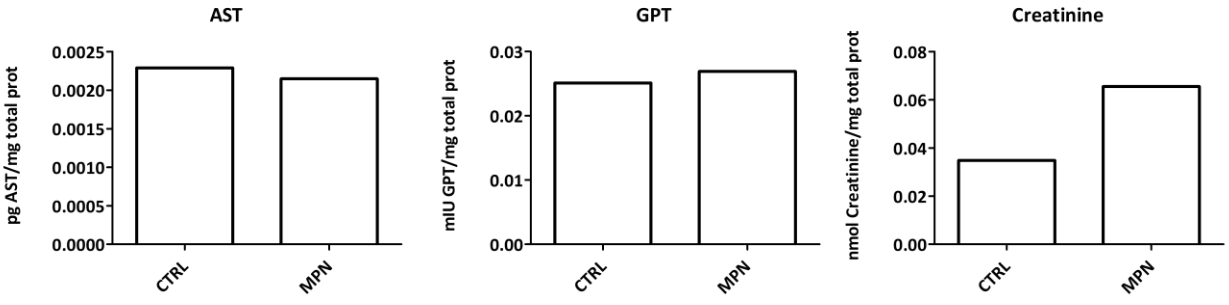

Supplementary Figure 7. Aspartate transaminase (AST), glutamic-piruvate transaminase (GPT) and creatinine levels in mice serum (ELISA test) (pool of 12 sera per point).

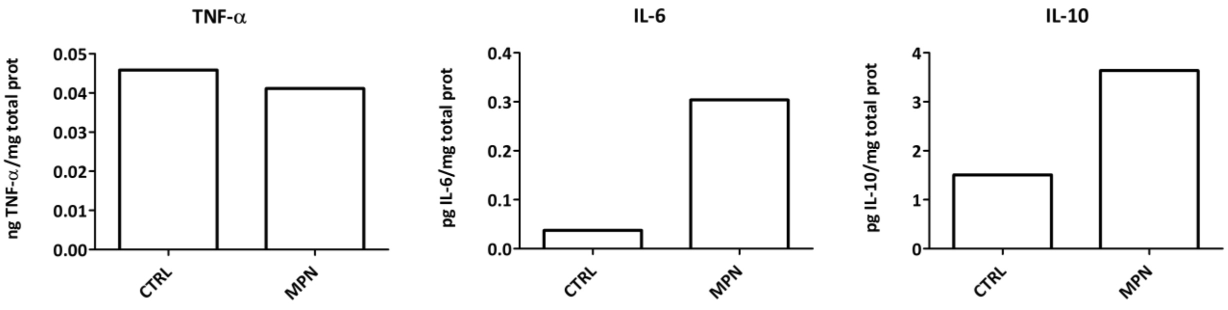

Supplementary Figure 8. Tumor Necrosis Factor-α (TNF-α), Interleukin-6 (IL-6), Interleukin-10 (IL-10) levels in mice serum (ELISA test) (pool of 12 sera per point).

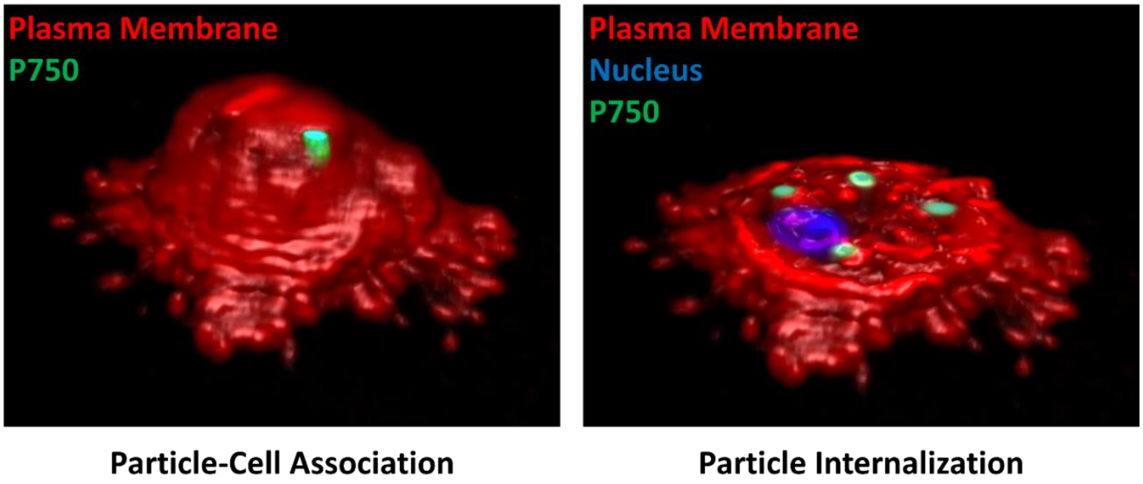

Supplementary Figure 9. 3D deconvolution of a z-stack representing the same cell. On the right, the whole z-stack series was reconstructed and an associated particle is shown on top of it. On the right a transversal cut was performed on the z-stack series allowing to expose the inside of the cell; it is possible to appreciate the presence of 4 internalized particles.

Statistical Analysis of Confocal Microscopy and Flow Cytometry Data. p-value tables of internalization analysis are obtained via ANOVA single test.

Confocal Microscopy Statistical Analysis (p value)					
	P750	rDPN	P200	SPN	LP
**- MP vs MP**	0.02	0.05	0.01	0.02	0.005

Flow Cytometry Statistical Analysis (p value)					
**CTRL vs:**	P750	rDPN	P200	SPN	LP
**0.05 mM**	0.007	0.00003	0.28	0.002	0.08
**0.15 mM**	0.01	0.0008	0.02	0.09	0.01
**0.25 mM**	0.003	0.00001	0.00001	0.006	0.007
**0.5 mM**	0.003	0.00003	0.004	0.0002	0.00002

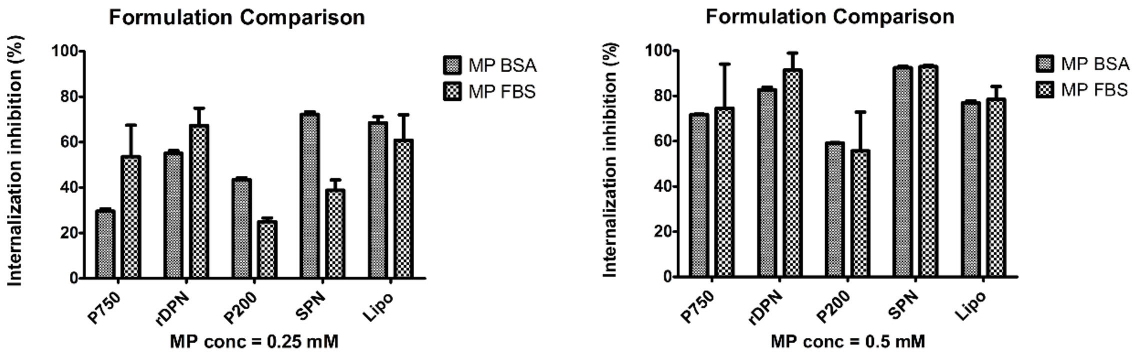

Supplementary Figure 10 Flow Cytometry analysis of particles internalization: comparison between MPN formulation prepared using MP + BSA or MP + FBS. (N=3)

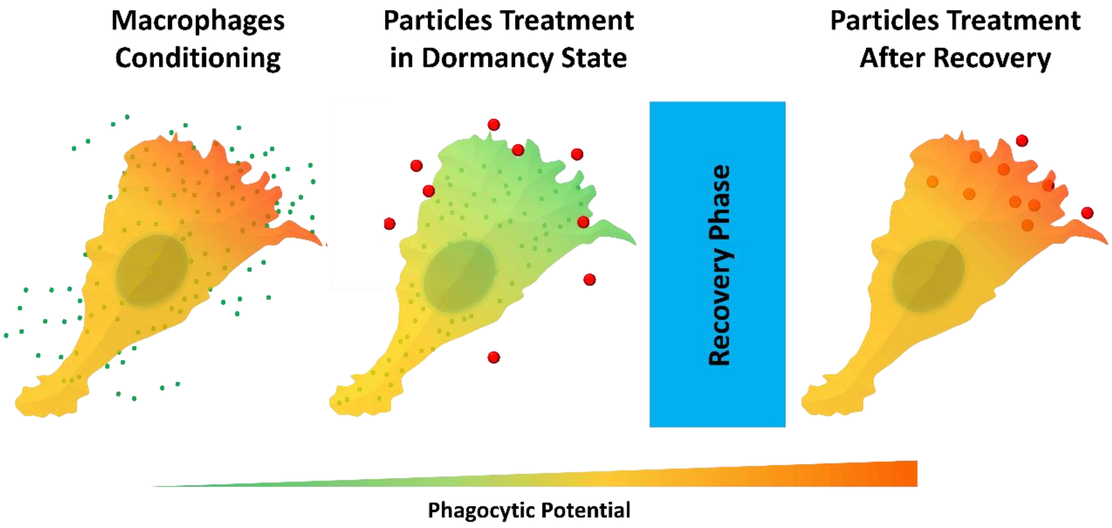

Supplementary Figure 11. Schematic representing the different outcomes obtained after macrophages conditioning with MPN in case the cells are treated with particles during the dormancy state (16h) or after a recovery phase (40h).

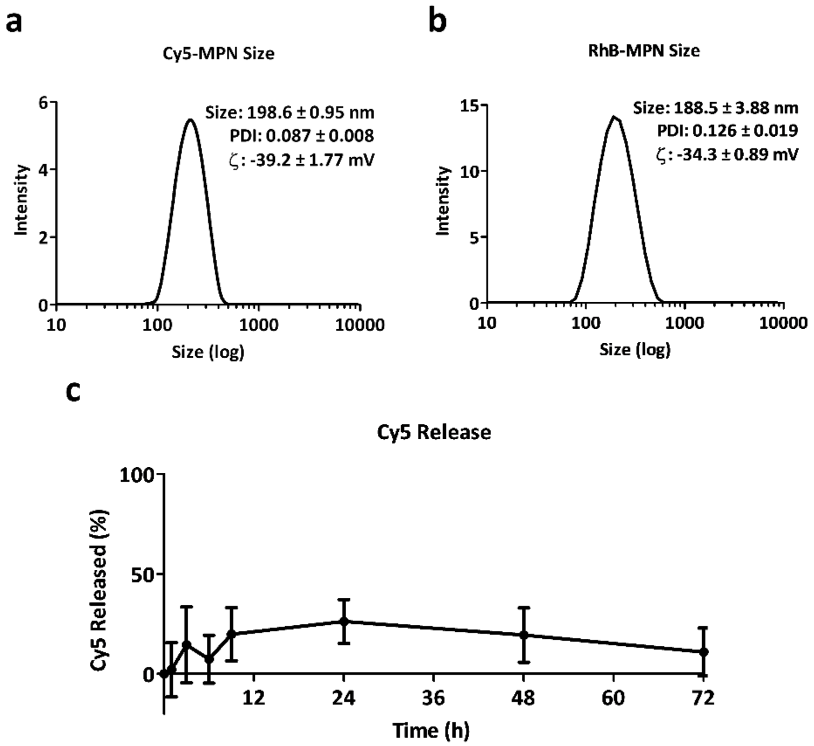

Supplementary Figure 12. a. Cy5-MPN size distribution and ζ-potential (DLS). **b.** Rhodamine-B-MPN size distribution and ζ-potential (DLS) **c.** Cy5 release over time.

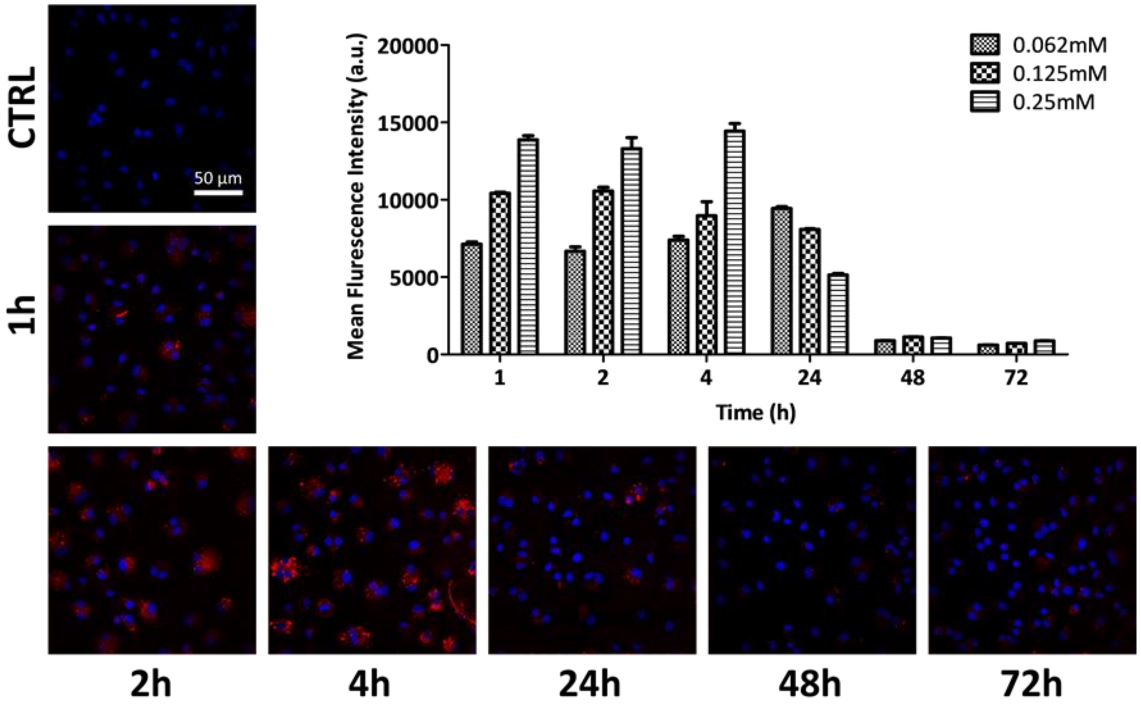

Supplementary Figure 13: Flow Cytometry (plot) and confocal microscopy images of BMDM treated with Cy5-MPN at different time points. Blue signal refers to nuclei (DAPI staining); red signal refers to particles.

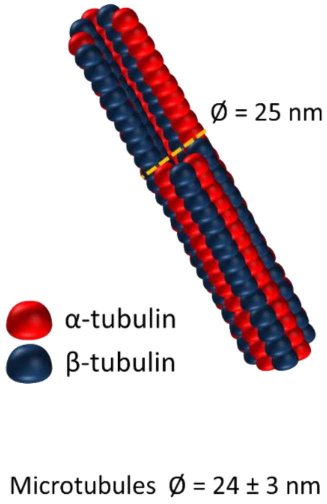

Supplementary Figure 14. Microtubule schematic.

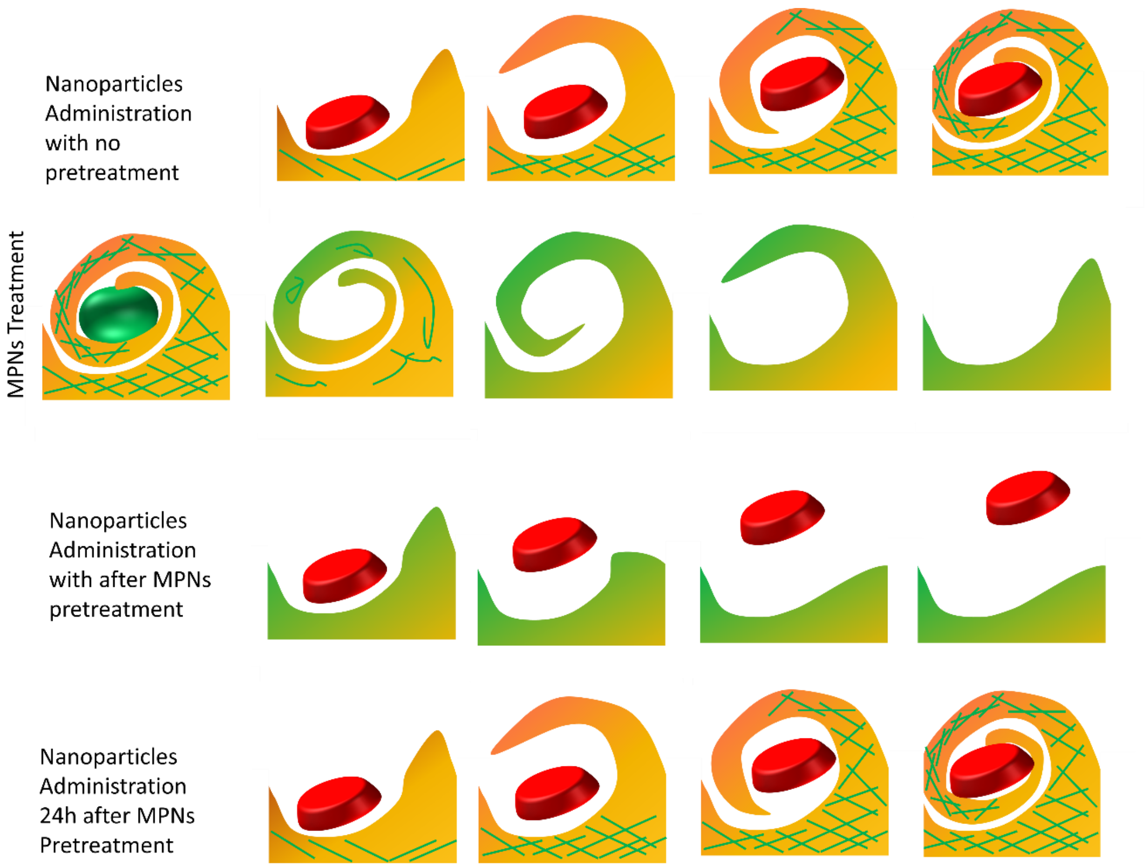

Supplementary Figure 15. Schematic of cell capability to rearrange microtubules: i) in case of no pretreatment with MPN (cells are only treated with nanoparticles and easily uptake them - first raw); ii) during MPN treatment (second raw); iii) during macrophages dormancy state (nanoparticles uptake is inhibited - third raw), and iiii) after a 24h recovery period (uptake function is fully restored and nanoparticles are again internalized - fourth raw)

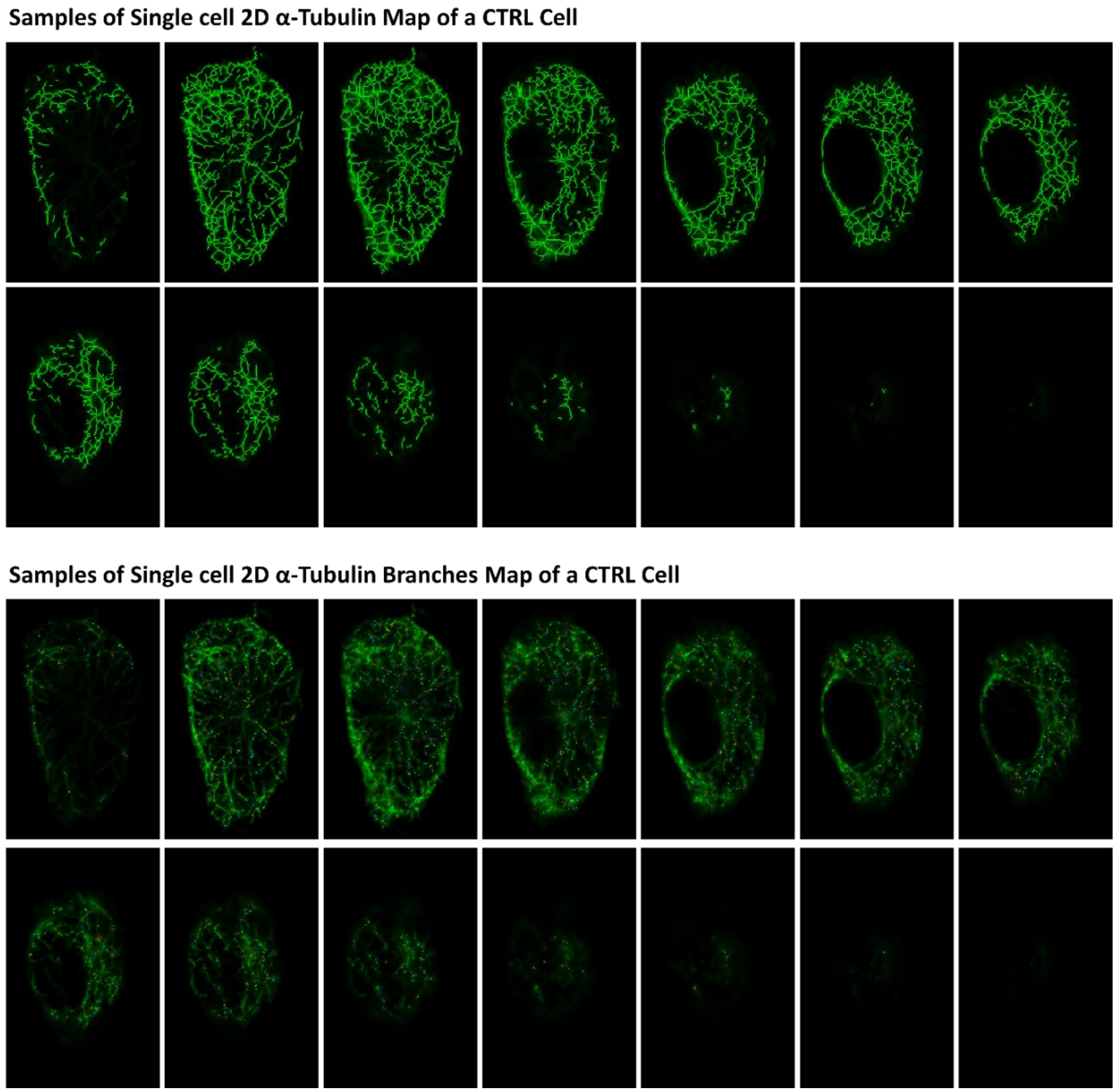

Supplementary Figure 16. Single cell 2D α-Tubulin Map and α-Tubulin branches map of a CTRL Cell.

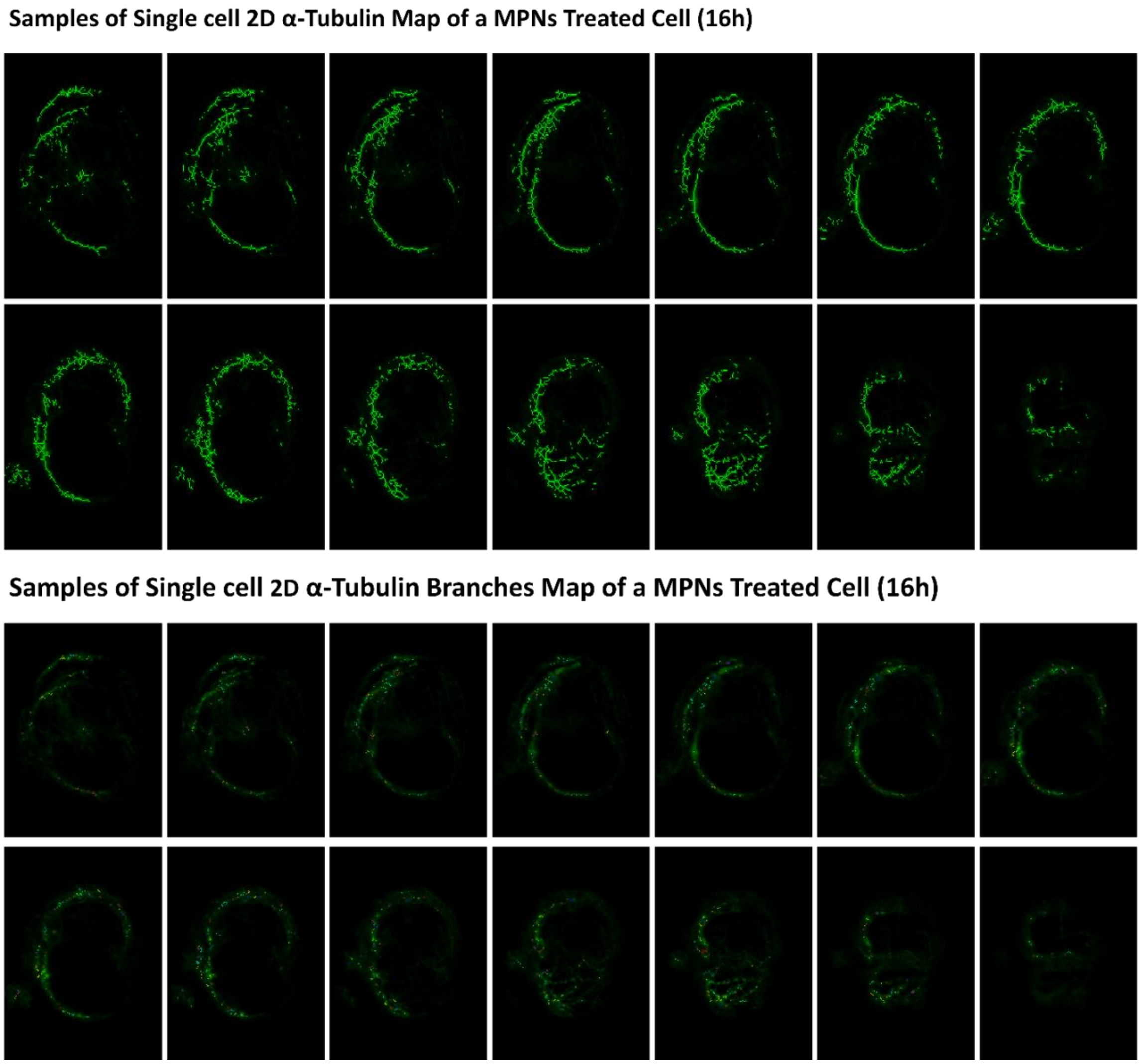

Supplementary Figure 17. Single cell 2D α-Tubulin Map and α-Tubulin branches map of a MPN treated Cell.

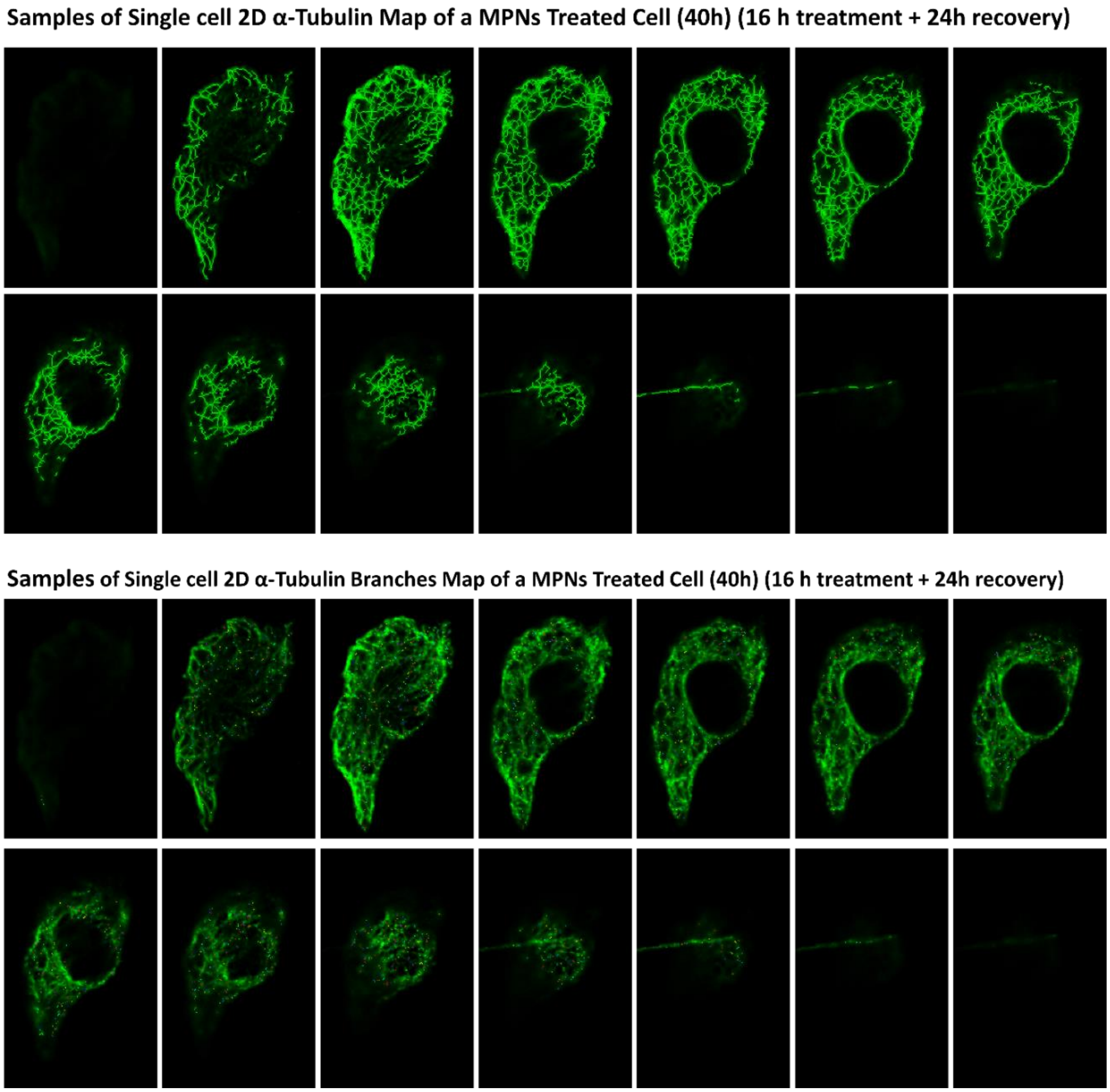

Supplementary Figure 18. Single cell 2D α-Tubulin Map and α-Tubulin branches map of a MPN treated cell which underwent a 24h recovery period.

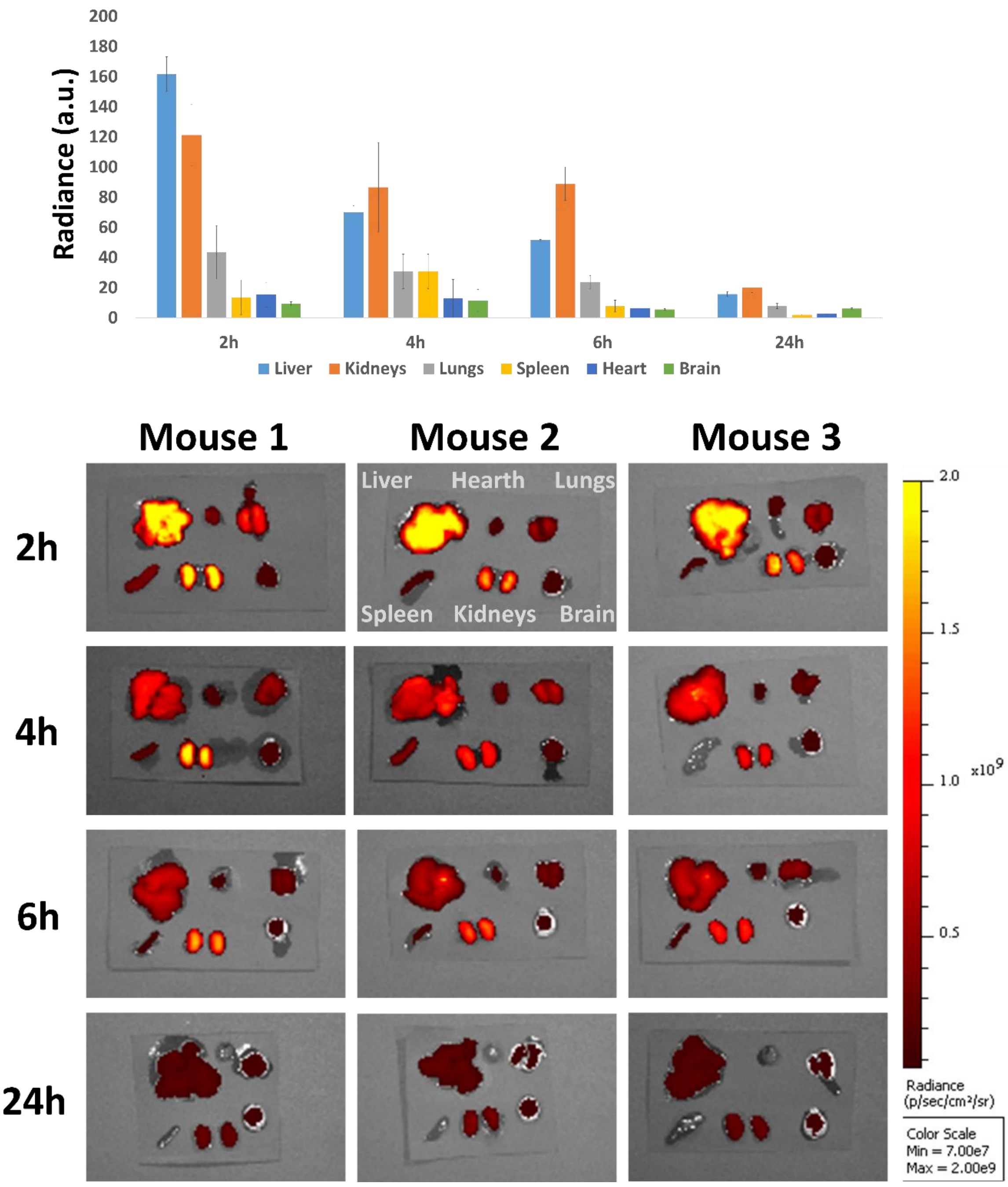

Supplementary Figure 19. Ex-Vivo biodistribution analysis of Cy5-MPN in C57BL/6J mice at different time points after MPN tail vein injection (methyl palmitate dose equivalent to 1.87 mg/ 20 g).

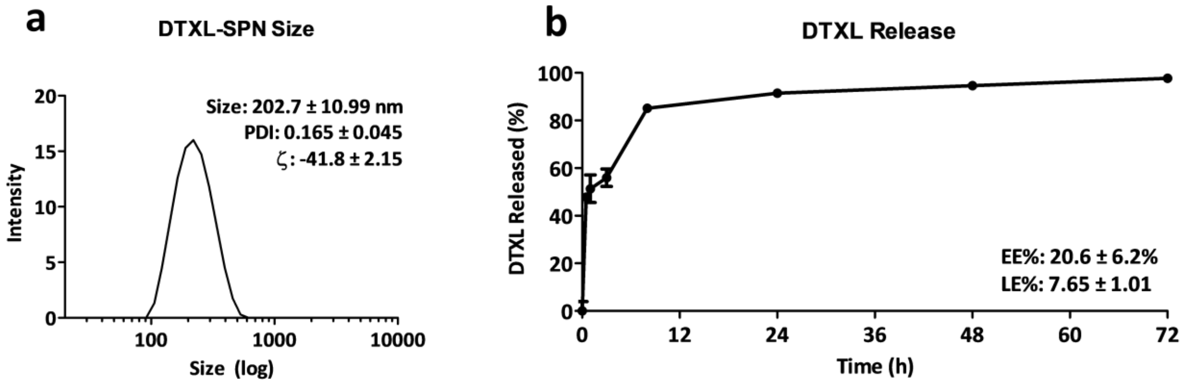

Supplementary Figure 20. a. DTXL-SPN size distribution and ζ-potential (DLS). **b.** DTXL release over time.

Statistical Analysis of U87-MG tumor treatment. Standard Deviation and P-values of tumor growth. Standard deviation of body weights.

**Tumor Growth - Standard Deviation**						
	Day 21	Day 23	Day 27	Day 30	Day 34	Day 37
**CTRL**	47.61	126.07	244.08	391.91	282.34	710.62
**DTXL-SPNs**	33.06	155.55	184.0485	383.11	447.45	825.01
**MPN + DTXL-SPNs**	27.75	79.21	120.37	302.37	341.26	234.31
**MPNs**	42.85	70.70	242.02	473.25	486.20	744.45

**Tumor Growth - P-value**				
	Day 27	Day 30	Day 34	Day 37
**CTRL** vs **SPNs**	0.882	0.867	0.776	0.942
**CTRL** vs **MPNs + DTXL SPNs**	0.067	0.063	0.002	0.014
**SPNs** vs **MPNs + DTXL SPNs**	0.071	0.043	0.022	0.051
**CTRL** vs **MPNs**	0.470	0.811	0.752	0.838

Body Weight - Standard Deviation						
	Day 21	Day 23	Day 27	Day 30	Day 34	Day 37
**CTRL**	0.87	1.16	1.30	2.13	2.25	2.53
**DTXL-SPN**	0.90	0.98	0.95	0.98	1.57	1.26
**MPN + DTXL-SPN**	2.36	2.27	1.28	1.78	2.12	2.39
**MPN**	2.15	2.45	1.99	2.04	2.47	2.17

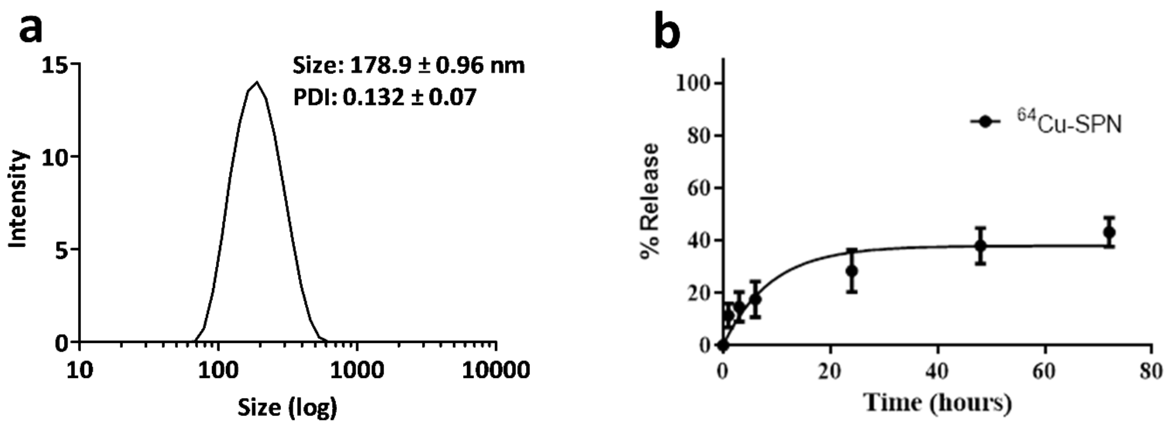

Supplementary Figure 21. a. ^{64}Cu-SPN size distribution (DLS). **b.** ^{64}Cu release over time.

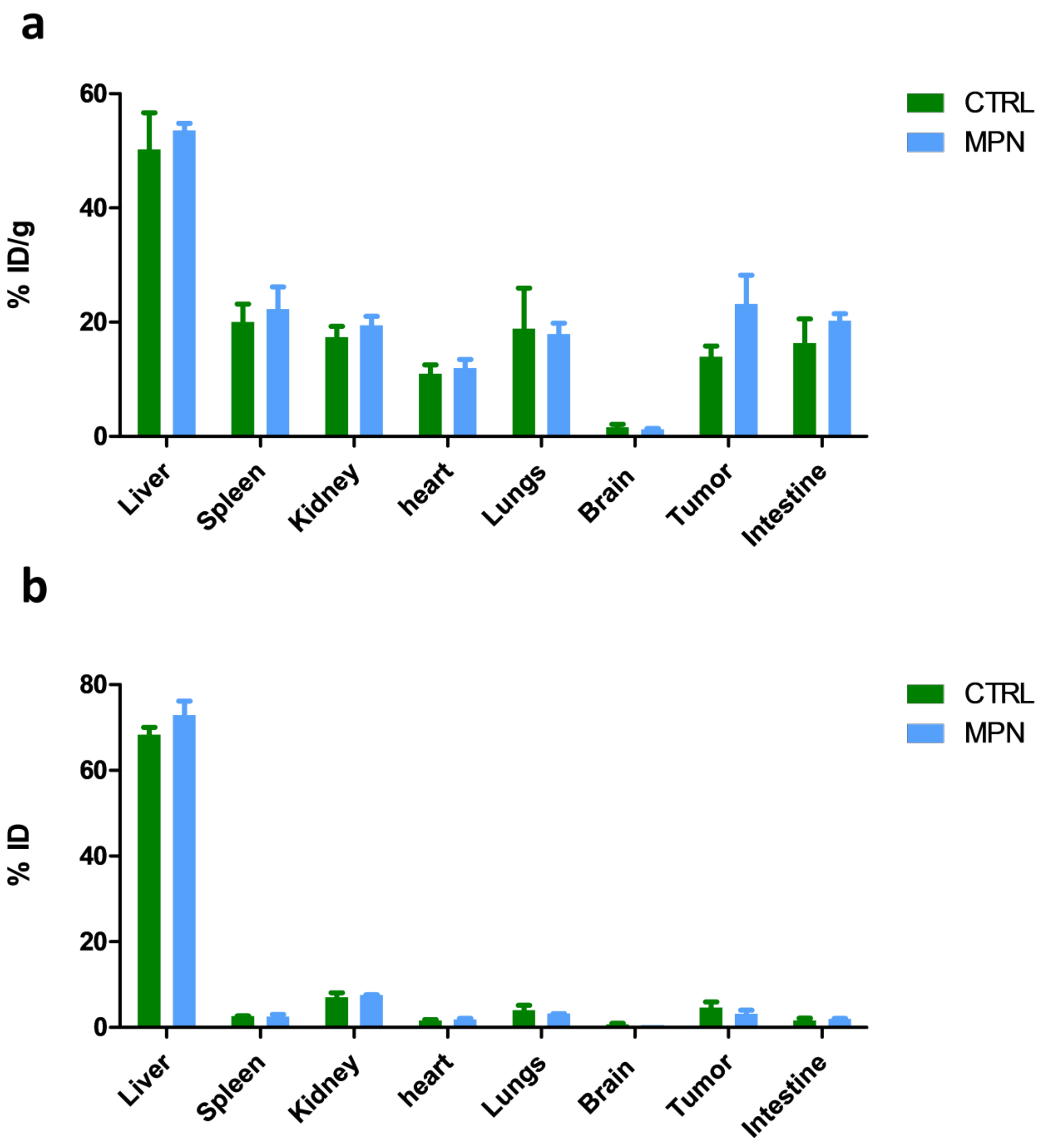

Supplementary Figure 22. ^{64}Cu-SPN biodistribution as **a.** percentage of the injected dose normalized by the organ mass (%ID/g); **b.** absolute percentage of the injected dose (%ID).
